# Multisensory integration in peripersonal space indexes consciousness states in sleep and disorders of consciousness

**DOI:** 10.1016/j.xcrm.2026.102705

**Published:** 2026-03-31

**Authors:** Tommaso Bertoni, Giulia Ricci, Jane Jöhr, Brunella Donno, Jacinthe Cataldi, Julia Fellrath, Aurelie Stephan, Carolina Foglia, Sandro Lecci, Floriane Dauvin, Marina Lopes Da Silva, Mattia Galigani, Polona Pozeg, Vincent Dunet, Marzia De Lucia, Jean-Paul Noel, Elisa Magosso, Karin Diserens, Francesca Siclari, Andrea Serino

**Affiliations:** 1 MySpace Lab, Department of Clinical Neurosciences, Lausanne University Hospital and University of Lausanne, Lausanne, Switzerland; 2 Translational Neural Engineering Lab, Neuro-X Institute, École Polytechnique Fédérale de Lausanne (EPFL), Lausanne, Switzerland; 3 Sleep and Dreams Group, Netherlands Institute for Neuroscience, Amsterdam, the Netherlands; 4 Acute Neurorehabilitation Unit, Department of Clinical Neurosciences, Lausanne University Hospital and University of Lausanne, Lausanne, Switzerland; 5 Center for Investigation and Research on Sleep, Lausanne University Hospital and University of Lausanne, Lausanne, Switzerland; 6 MANIBUS Lab, Psychology Department, University of Turin, Turin, Italy; 7 Department of Medical Radiology, Lausanne University Hospital and University of Lausanne, Lausanne, Switzerland; 8 Laboratoire de Recherche en Neuroimagerie (LREN), Lausanne University Hospital and University of Lausanne, Lausanne, Switzerland; 9 Department of Neuroscience, University of Minnesota, Minneapolis, MN, USA; 10 Department of Electrical, Electronic, and Information Engineering “Guglielmo Marconi”, University of Bologna, Cesena Campus, Cesena, Italy

**Keywords:** consciousness, disorders of consciousness, sleep, peripersonal space, multisensory integration, beta oscillations, EEG

## Abstract

Reliably detecting consciousness in unresponsive patients remains an urgent ethical and clinical challenge, as no behavior-independent marker is currently accepted in clinical practice. We characterize consciousness as linked to a representation of the embodied subject of experience, mediated by multisensory integration within the peripersonal space (PPS) system. We test whether a neural marker of PPS representation could detect consciousness and predict clinical outcome in disorders of consciousness (DoC) patients. Using high-density electroencephalography (EEG) during a task-free audiotactile task, we derive a PPS index based on high-beta oscillations. In healthy participants, the PPS index is present during wakefulness and dreaming, but absent in dreamless sleep. In 72 DoC patients, the PPS index correlates with behavioral measures of consciousness and predicts recovery at discharge. The index is associated with forebrain mesocircuit integrity. These findings highlight a bedside-compatible electrophysiological marker with potential clinical utility for detecting covert consciousness and predicting outcomes in non-responsive patients.

## Highlights

• Beta centro-parietal oscillations reflect peripersonal space (PPS) representation
• Such PPS representation index detects consciousness during wakefulness and sleep
• The PPS index predicts clinical outcome in disorders of consciousness patients
• The PPS index correlates with the integrity of the forebrain mesocircuit

## Graphical abstract

**Figure:**
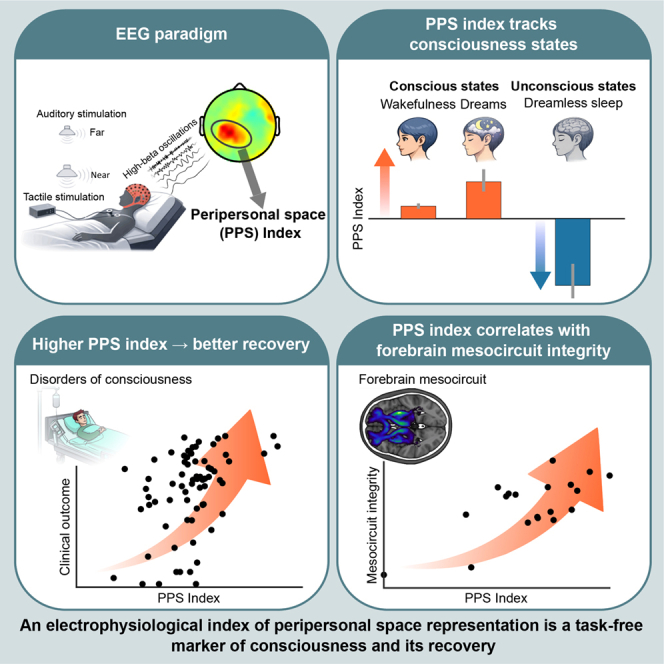

## Teaser

Bertoni et al. show that oscillations in neural electrical activity support the representation of the space near the body, linked to the experience of being “here and now.” The same oscillations detect the presence of consciousness during wakefulness and sleep and predict patients’ chances of recovery from coma.

## Introduction

The quest to identify the neural correlates of consciousness has fueled decades of research and debate.^1^^,^^2^^,^^3^ Yet, despite substantial theoretical progress, clinical translation remains limited—especially in unresponsive patients. In such cases, behavioral examination alone is insufficient to establish whether consciousness is present, posing critical diagnostic, ethical, and therapeutic challenges.^4^ Addressing this gap requires objective, physiology-based markers that do not rely on overt responses and can generalize across healthy and pathological states.

While most theories of consciousness focus on mechanisms underlying external stimulus awareness,^5^ neurophenomenological approaches also explore the structure of consciousness, emphasizing the subjective experience of the self within its environment.^6^^,^^7^ This self-representation, termed minimal selfhood,^8^ arises from the integration of multisensory and motor information about the body and its surroundings, leading to the concepts of Bodily Self-Consciousness and Bodily Self.^9^^,^^10^^,^^11^

A critical neural system integrating bodily cues with external stimuli supporting the Bodily Self is the peripersonal space (PPS) system. PPS is constituted by a network of premotor and parietal regions that integrates tactile information on the body with external—visual or auditory—stimuli as a function of their distance, in a body-centered reference frame.^12^^,^^13^ This way, PPS builds a representation of the body in a space of potential interactions, constituting a dynamic interface between the self and its environment. Multimodal evidence^14^^,^^15^^,^^16^^,^^17^^,^^18^ supports the view that PPS is involved in integrating multisensory stimuli underlying Bodily Self-Consciousness. Classic paradigms^19^^,^^20^^,^^21^^,^^22^^,^^23^ like the rubber hand and full-body illusions demonstrate how altering the spatiotemporal coherence of multisensory stimuli in PPS influences Bodily Self-Consciousness. Accordingly, PPS representation varies following changes in Bodily Self-Consciousness.^15^^,^^17^^,^^24^ Importantly, alterations in PPS representation have been linked to disorders of consciousness (DoCs), allowing distinction between patients suffering from a true DoC from patients with a phenotype of clinical cognitive motor dissociation (c-CMD).^25^^,^^26^ Here, we use the term c-CMD to explicitly distinguish a clinically defined phenotype, identified at bedside using the Motor Behavior Tool-Revised (MBT-r), from neuroimaging-based CMD.^25^

Building on this evidence, we hypothesized that PPS processing may serve as a task-free neural marker of minimal selfhood, and thereby of consciousness itself, which can be applied to unresponsive patients exhibiting a DoC behavioral phenotype.

**Figure 1:**
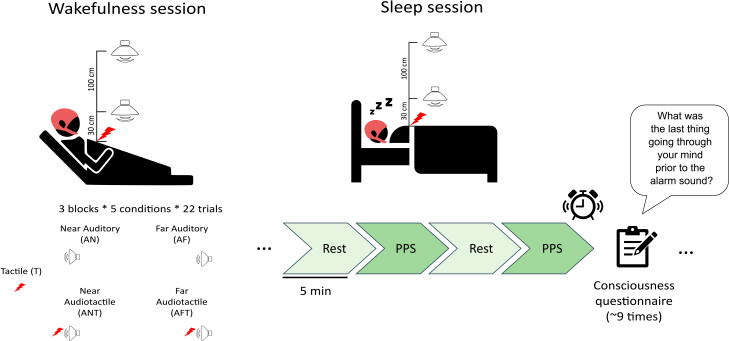
Sleep experiment design The 5 experimental conditions are shown on the left. In the wakefulness session, 3 blocks of 110 stimuli (22 per condition, randomized) were administered (PPS paradigm). In the sleep session, participants received 5-min blocks with stimuli from the PPS paradigm as in the wakefulness session, alternated with 5-min blocks of resting state. About 9 times per night, they were awakened and responded to a questionnaire about the presence and the content of conscious experience before the awakening; 256-channel EEG was recorded throughout the experiment.

To this end, we first developed and tested a neurophysiological marker of PPS processing in healthy individuals across the sleep-wake cycle. Sleep offers a natural model of fluctuation in consciousness, ranging from unconscious (dreamless) to conscious (dreaming) states. Using high-density electroencephalography (EEG) and a well-established audiotactile stimulation paradigm (Figure 1),^27^^,^^28^^,^^29^^,^^30^^,^^31^ we identified a robust PPS index—characterized by high-beta oscillatory activity over centroparietal regions—elicited by stimuli near vs. far from the body. This PPS index was consistently present during wakefulness and reliably distinguished dreaming (conscious) from dreamless (unconscious) sleep. These results validate our PPS index as a marker of consciousness that is independent of behavior and task performance.

We then applied this marker to a large cohort of patients with DoCs (*n* = 72), collected over a 5-year multimodal assessment study. We found that the PPS index was associated with behavioral evidence of consciousness and, importantly, predicted patients’ clinical outcome at discharge, improving predictions based on behavioral clinical scales. Finally, we investigated the neural underpinnings of PPS processing in this patient cohort, revealing associations with the structural integrity of the forebrain mesocircuit and with the decoupling between the default mode network (DMN) and dorsal attention network (DAN). These findings link PPS representation to the presence of consciousness and to the broader functional architecture supporting recovery from DoCs.

## Results

### High-beta oscillations index PPS representation during wakefulness

We first identified a neurophysiological marker of PPS representation in 15 healthy awake participants by examining space-dependent multisensory effects. Accordingly, the PPS index was calculated as the difference in spectral power for near-far unisensory stimuli minus the difference for near-far multisensory stimuli, targeting interactions between stimulus distance and sensory modality (see STAR Methods and Noel et al.^29^ and Ronga et al.^31^).

**Figure 2:**
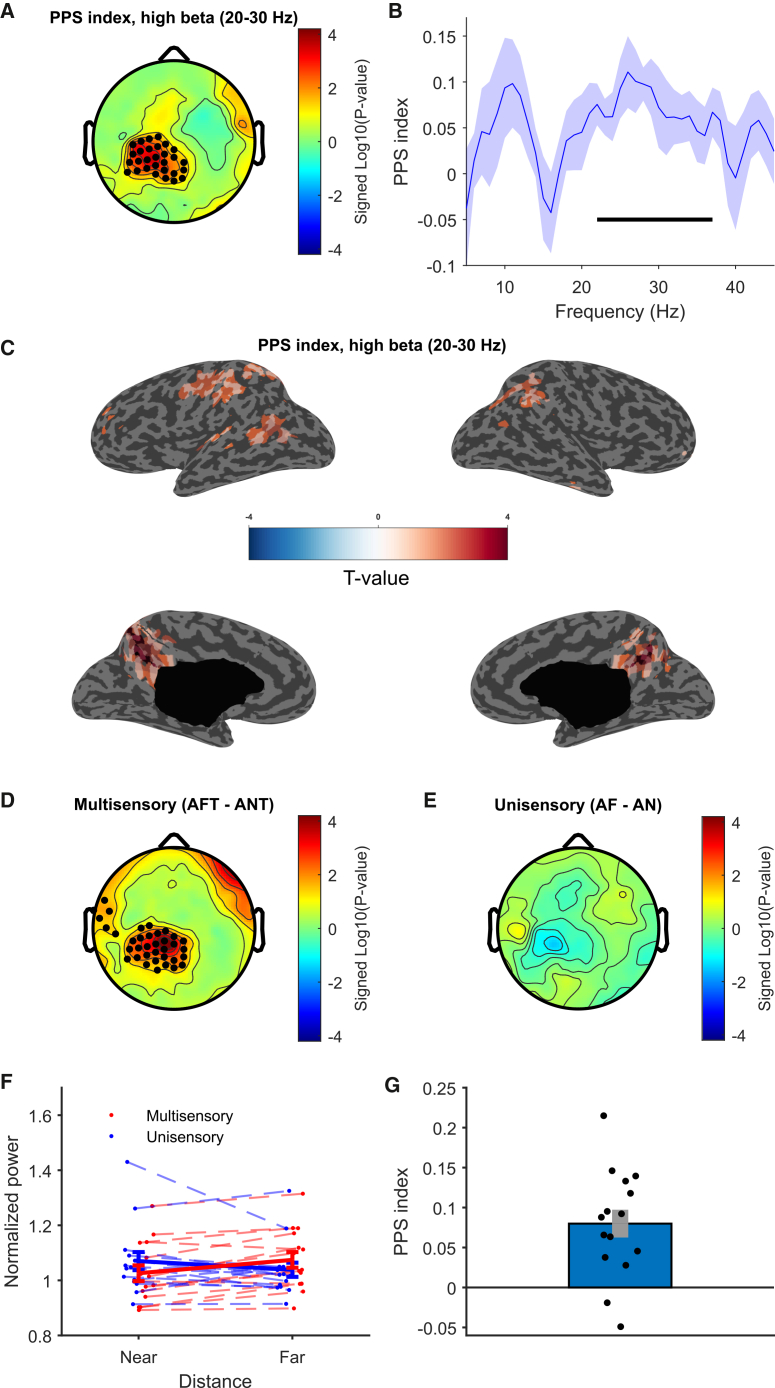
Spectral index of PPS representation during wakefulness (A) Topography of the PPS index in the high-beta (20–30 Hz) frequency range. Black dots in the centro-parietal area denote electrodes belonging to the significant cluster. (B) PPS index by frequency in the significant cluster. Shades indicate standard errors, and the horizontal line indicates frequencies where the PPS index is significantly greater than zero. (C) Cortical reconstruction of the PPS index, thresholded at *p* < 0.05. (D) Comparison between near and far multisensory stimuli (audiotactile far [AFT]-audiotactile near [ANT]). Black dots denote electrodes belonging to the significant cluster. (E) Topography of the near-far comparison within unisensory stimuli (audio far [AF]-audio near [AN]). (F) Power in the high-beta band within the cluster identified in (A) in the four experimental conditions for each participant. Thicker lines represent means, and error bars represent standard errors. (G) PPS index in the high-beta cluster for each participant (the error bar represents the standard error).

We computed the PPS index across five frequency bands (theta, alpha, low beta, high-beta, and gamma) during the 1-s post-stimulus period for each channel and tested it against zero. We found one significant cluster in centroparietal electrodes (*p* = 0.0084, see STAR Methods) indicating PPS processing in the high-beta (20–30 Hz) range (Figure 2A). We then studied the frequency of the PPS effect in detail within this cluster, by computing the PPS index for all frequencies. This revealed that the PPS index was significantly different from zero between 22 and 37 Hz, peaking at 26 Hz, thus mainly covering the high-beta band (Figure 2B). Restricting the analysis to such frequency range yielded comparable results to the high-beta band, as shown in Figure S1.

Source reconstruction localized significant PPS-related activations to the bilateral precuneus, cingulate, intraparietal sulcus, left superior parietal lobule, right inferior parietal lobule, left sensorimotor cortex, and small clusters in the superior temporal sulcus, temporo-parietal junction, and superior frontal gyrus (Figure 2C). These regions align with PPS-related areas identified in prior neuroimaging studies.^18^

To further validate the PPS effect, we compared responses to multisensory (audiotactile near [ANT] vs. audiotactile far [AFT]) and unisensory (audio near [AN] vs. audio far [AF]) stimuli as a function of distance. As predicted by theory, a significant cluster was found for multisensory stimuli (*p* = 0.0088; Figure 2D), but not for unisensory stimuli (Figure 2E). Post hoc analysis within the significant interaction cluster revealed stronger high-beta desynchronization (power reduction) for near vs. far audiotactile stimuli (*p* < 0.001; Figure 2F). No significant differences were observed for unisensory stimuli (*p* = 0.14).

Finally, the high-beta PPS index was consistently positive in all but two participants (Figure 2G), indicating a robust space-dependent modulation of multisensory processing as the driver of the PPS effect. Still, possibly due to random variability or individual differences in oscillatory activity, 2 of 15 subjects showed a negative PPS index. Restricting the analysis to the frequency band with the strongest effects (25–30 Hz) yielded a positive PPS index for all subjects (see Figure S2).

### Spectral markers of PPS representation characterize conscious experience during sleep

**Figure 3:**
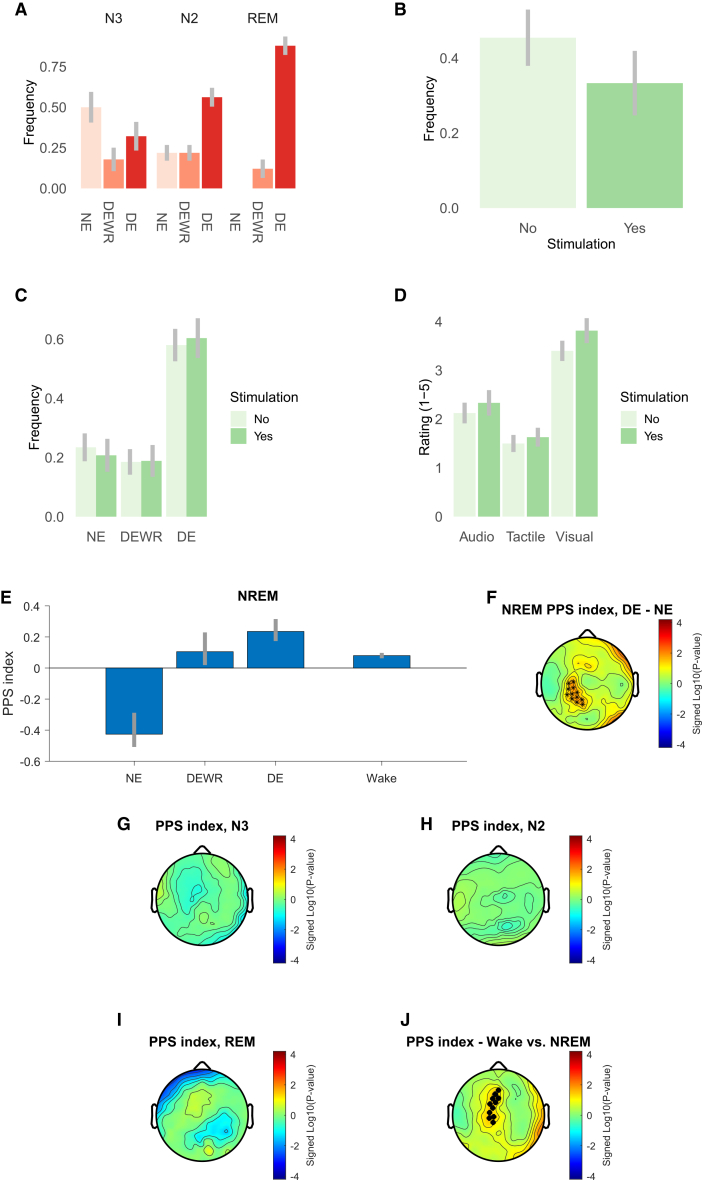
PPS index across sleep stages and reported consciousness states (A) Frequency of no experience (NE), dream experience without recall (DEWR), and dream experience (DE) reports depending on the sleep stage. Vertical bars indicate standard errors. (B) Frequency of reports of a link between sensory content of the dream and experimental stimulation, as a function of the presence of experimental stimulation. Vertical bars indicate standard errors. (C) Frequency of NE, DEWR, and DE reports depending on the presence or absence of experimental stimulation before the awakening. Vertical bars indicate standard errors. (D) Average rating for the presence of auditory, tactile, or visual sensations depending on the presence or absence of stimulation. Vertical bars indicate standard errors. (E) PPS index in the high-beta cluster (identified during wakefulness) depending on the reported consciousness state at awakening, i.e., unconscious (NE), conscious without recall (DEWR) or conscious with recall (DE). The PPS index during wakefulness is also shown for comparison. Error bars show 66% confidence intervals obtained by bootstrapping. (F–I) Contrast of PPS index in the DE vs. NE conditions. Asterisks indicate electrodes that are significant in this contrast and also belong to the awake high-beta cluster. (G–I) The PPS index in the N3, N2, and REM sleep phases, respectively. (J) Contrast for the PPS index between wakefulness and NREM sleep. Black dots indicate electrodes belonging to the significant cluster.

To validate the PPS index as a consciousness marker, we investigated whether the high-beta PPS index identified during wakefulness could distinguish conscious from unconscious states during sleep. Consciousness states were measured via a serial awakening protocol,^32^ with dreaming where content could be recalled (DE) and no-dreaming (NE) periods determined by subjective reports. Consistent with previous findings, the prevalence of DE reports increased across sleep stages from N3 (32.1%) to N2 (56.2%) and rapid eye movement (REM) (87.9%), while NE reports followed the opposite trend, decreasing from N3 (50%), to N2 (21.9%) and REM (0%) (ꭓ^2^ = 26.3, *p* < 0.0001, Figure 3A). The presence of experimental stimuli did not significantly influence reported consciousness state (ꭓ^2^ = 0.136, *p* = 0.934) or sensory content (visual, auditory, tactile stimuli) in dreams (all *p* > 0.21), indicating that dream content was internally generated (Figures 3B–3D).

To test the PPS index’s relationship with consciousness, we analyzed EEG responses in the 20 s preceding awakenings during non-REM (NREM) (N2+N3) sleep.^33^ Due to the absence of NE reports in REM sleep, the analysis was only performed in NREM (N2+N3) sleep.^34^^,^^35^ In DE trials, the PPS index was significantly higher than in NE trials (*p* = 0.026) and comparable to wakefulness (*p* = 0.43). In contrast, the PPS index for NE trials was lower than both DE (*p* = 0.026) and wakefulness (*p* = 0.03, Figure 3E; see Figures S3–S4 for additional plots). These differences could not be explained by a lack of evoked activity, as robust responses were observed in NE trials compared to pre-stimulus baselines (see Figures S5–S6). Additionally, there was no significant difference in the PPS index between DE and dreaming experience without recall (DEWR; *p* = 0.67), ruling out recall-related confounds.

We validated this finding by directly comparing the DE-NE difference in PPS index across the entire topography, revealing significant overlaps with the high-beta cluster identified during wakefulness (9 of 29 channels; Figure 3F). Finally, we assessed PPS effects independent of consciousness state across sleep stages. No significant clusters emerged in N3, N2, or REM sleep (Figures 3G–3I). However, as expected, the PPS index was lower in NREM sleep compared to wakefulness (*p* = 0.035; Figure 3J). A trend for higher PPS index in REM compared to N3 sleep was observed in fronto-central channels, but did not survive correction, likely due to the limited number of REM trials (see Figure S7).

Taken together, these results show a neural marker of PPS representation emerged in conscious states, i.e., during wakefulness and dreams, and vanished during unconscious sleep, suggesting that the PPS index allows to identify the presence of consciousness irrespective of behavioral output.

### Spectral markers of PPS representation index consciousness levels and predict clinical outcome in patients with DoC behavioral phenotype

We then tested whether the PPS index, besides indexing consciousness in healthy individuals, could be applied to detect residual consciousness and predict clinical outcome through a bedside examination in 72 patients with a DoC behavioral phenotype. The presence of residual consciousness in the acute phase, even when not detectable through routine clinical examination (covert consciousness^36^), is possibly linked with higher chances of positive long-term outcome.^37^ We thus hypothesized that the PPS index, being independent of patients’ behavioral responses, could also detect covert consciousness, predicting patients' outcome beyond standard clinical assessments.

Seventy-two patients in an acute neurorehabilitation unit (mean 31.7 days post-injury) underwent EEG recordings adapted from the healthy participant paradigm to a bedside setting (see STAR Methods). Patients were assessed using the Coma Recovery Scale-Revised (CRS-R) and the MBT-r, with 67 diagnosed as c-CMD and 5 as true DoC (see STAR Methods and Table S1). Clinical outcomes at discharge (∼23.5 days later) were assessed by expert clinicians using a composite index of scales measuring motor, functional, and cognitive recovery (outcome index, see STAR Methods and Pozeg et al.^38^).

**Figure 4:**
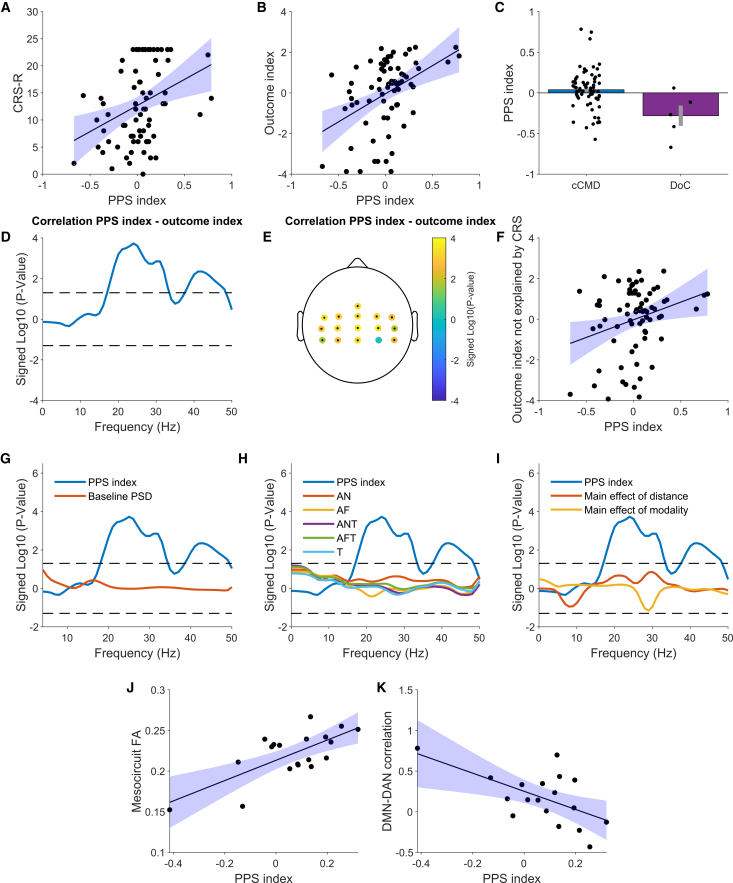
PPS index, consciousness, and clinical outcome in patients with a DoC behavioral phenotype (A) Correlation between PPS index (high-beta, average on the 16 channels) and CRS-R at recording time. (B) Correlation between PPS index and our composite clinical outcome index at discharge. (C) PPS index in patients who were diagnosed with c-CMD and true DoC according to the motor behavior tool revised MBT-r. Error bars represent standard errors. (D) *p* values for the correlation between PPS index (average on 16 channels) and outcome index at the frequency level. Positive values indicate a positive correlation, and vice versa for negative. Dashed lines indicate the significance threshold *p* = 0.05. (E) Correlation between high-beta PPS index and outcome index at the single-channel level for all the channels in the clinical EEG setup. Black dots indicate significant channels at *p* < 0.05. (F) Correlation between PPS index and outcome index, after regressing out CRS-R scores at the time of the recording, i.e., the amount of variability in the outcome index not explained by the CRS-R score at the time of assessment. (G–I) *p* values from the same analysis as in B but comparing the PPS index with other potential predictors of clinical outcome: (G) PPS index vs. power spectral density during the 0.5-s pre-stimulus baseline period; (H) PPS index vs. high-beta power in all the individual experimental conditions (tactile, T; audio-near, AN; audio-far, AF; audio-tactile near ANT; audio-tactile far, AFT); (I) PPS index vs. the main effect of distance and modality. (J) Correlation between PPS index and forebrain mesocircuit integrity (fractional anisotropy, FA). (K) Correlation between PPS index and DMN-DAN connectivity.

Since the clinical 16-channel EEG setup covers centro-parietal, sensorimotor areas, largely corresponding to the cluster of high-beta modulation found in the healthy awake participants, we averaged the high-beta PPS index across the 16 channels. The PPS index correlated significantly with CRS-R scores collected at the time of the EEG recording (R = 0.35, *p* = 0.002) and was higher in c-CMD patients (*p* = 0.019; Figures 4A and 4C), confirming the link between PPS representation and consciousness levels in patients with DoC.^29^ Crucially, the PPS index predicted the outcome index at discharge (R = 0.43, *p* = 0.0001; Figure 4B), outperforming its correlation with CRS-R. Patients with more positive PPS index (i.e., in the direction of healthy awake participants) demonstrated a better recovery. These results were not driven by smaller or absent responses in patients with a worse outcome, since evoked responses to the stimulation in general were present irrespectively of the outcome (see Figure S8). Spectral analysis confirmed that the correlation between the PPS index and the outcome index was specific to the high-beta band (18–33 Hz, peaking at 24 Hz; Figure 4D). Restricting our main analysis (PPS index-outcome index correlation) to the significant frequency range only slightly improved the results (R = 0.44, *p* = 0.0001), further justifying the use of the high-beta frequency range. Channel-level analysis revealed significant correlations across all but one channel, with stronger effects on the left side (Figure 4E).

We then tested whether the PPS index could predict the clinical outcome beyond standard clinical information (CRS-R) available at the time of the recording. We thus tested the correlation between the PPS index and the outcome index while adding the CRS-R at the time of the recording as a covariate. The correlation between the PPS index and the outcome index remained significant (*p* = 0.021). To rule out that this may be simply because of the ceiling effect in the CRS-R, which saturates at 23 and is less suitable to capture differences in patients emerging from DoC, we repeated the analysis after removing patients with a CRS-R of 23 and still obtained significant results (*p* = 0.044). The analysis was significant after removing patients with CRS-R values of 20 or more (see Figure S9). To visualize this result, we regressed out from the outcome index the variability explained by the CRS-R. The residual variability in the outcome index not explained by CRS-R significantly correlated with the PPS index (R = 0.25, *p* = 0.03, Figure 4F), indicating that the PPS index provides complementary information about clinical outcomes beyond what CRS-R captures.

The PPS index also outperformed simpler EEG features, including baseline power spectra, responses to individual experimental conditions, and main effects of distance or modality, in predicting the outcome index (Figures 4G–4I).

To further assess the clinical value of these results, we used the PPS index to perform a binary classification of clinical outcomes (favorable vs. unfavorable), and benchmarked its performance against the standard behavioral evaluation provided by the CRS-R. Clinical outcome was defined using the Disability Rating Scale (DRS), categorizing patients with scores from 0 to 6 (no to moderate disability; 23 of 72 patients) as favorable outcomes and those scoring 7 to 29 (moderately severe disability to extreme vegetative state; 49 patients) as unfavorable. Following an approach similar to Kustermann et al.,^39^ we identified classification thresholds for both the PPS index and CRS-R to maximize the positive predictive value (PPV), while ensuring that at least 10% of the patients were positively classified. As shown in Table 1, the PPS index achieved superior PPV and specificity (0.86 and 0.98, respectively) compared to the CRS-R (0.67 and 0.89, respectively). Future studies may refine threshold identification depending on the specific cohort and EEG setup and define individualized frequency bands to improve these predictions.

**Table 1:** Comparison of outcome prediction metrics for CRS-R, PPS index, and their combination

Combined	CRS-R	PPS	Combined
True-positives	12	6	8
False-positives	6	1	0
False-negatives	7	13	11
True-negatives	47	52	53
PPV	0.67	0.86	1
NPV	0.87	0.8	0.83
Sensitivity	0.63	0.32	0.42
Specificity	0.89	0.98	1
Accuracy	0.82	0.81	0.85

True-positive indicates patients with a favorable outcome correctly predicted to have a favorable outcome (DRS of 6 or lower).

We argue that the principal added value of the PPS index lies in its contribution to multimodal assessments of DoCs, which are increasingly recognized as critical for accurate diagnosis and prognosis.^40^^,^^41^ Thus, to evaluate the benefit of combining behavioral and neurophysiological markers, we assessed the predictive performance of a joint model including both the PPS index and CRS-R. To obtain the combined index, the PPS index and CRS-R were normalized through *Z* score and then summed. This multimodal approach yielded perfect PPV and specificity (both 1.00) and higher overall accuracy (0.85) than the CRS-R scale alone.

Structural and functional neuroimaging markers were then examined in a subset of 19 patients with magnetic resonance imaging (MRI) data.^38^ We first examined a marker of structural integrity (fractional anisotropy) of the anterior forebrain mesocircuit, principally composed of frontal cortical regions, thalamus, and striatum, that has been shown to correlate with the severity of DoC and recovery potential.^42^^,^^43^ This index was positively correlated with the PPS index (R = 0.71, *p* = 0.0006, Figure 4J), indicating that patients with greater structural integrity in the mesocircuit also had a higher PPS index. Additionally, the PPS index was negatively correlated with the coupling between the activity of the DMN and the DAN^42^: patients with a higher PPS index also showed a stronger anti-correlation between DMN and DAN (R = −0.59, *p* = 0.01; Figure 4K). The DMN-DAN anticorrelation is robustly present in healthy awake participants,^44^ and its absence characterizes the severity of DoC.^45^^,^^46^ Further analyses showed that DMN-DAN coupling and mesocircuit integrity were also highly correlated between them (R = 0.74). To disentangle the crossed dependencies between these factors, we conducted a multiple regression predicting the PPS index from both DMN-DAN anticorrelation and mesocircuit integrity. When accounting for the shared variance between these two factors, only mesocircuit integrity, and not DMN-DAN anticorrelation, still predicted the PPS index (*p* = 0.031 vs. *p* = 0.66).

Finally, we explored whether the PPS index could be further combined with these MRI-based indexes, to investigate the potential for our index to be integrated in a battery of clinical and paraclinical tests to predict the recovery from DoC. As in our previous analysis (see Table 1), we defined patients with a DRS between 0 and 6 as positive outcomes (7 of 19) and defined classification thresholds on mesocircuit integrity, PPS index, and their combination. Due to the small sample of patients in the MRI cohort, we had to modify the criterion for threshold definition with respect to our previous analysis. We still defined the threshold to maximize the PPV, but set the minimum percentage of positively classified patients to 20% instead of 10% of the total (which would result in only 2 patients). Thus, the current analysis cannot be directly compared with the previous analysis. Again, the PPS index yielded a relatively high PPV, negative predictive value (NPV), and specificity, at the expense of a lower sensitivity. Importantly, when combined with the PPS index and the mesocircuit integrity, the predictive power compared to the PPS index alone increased or remained constant in all metrics, suggesting that the PPS index is best used in combination with other indicators of DoC recovery (see Table 2).

**Table 2:** Comparison of outcome prediction metrics for PPS index and its combination with mesocircuit integrity

	PPS	PPS + Mesocircuit
True-positives	3	4
False-positives	1	1
False-negatives	4	3
True-negatives	11	11
PPV	0.75	0.8
NPV	0.73	0.79
Sensitivity	0.43	0.57
Specificity	0.92	0.92
Accuracy	0.74	0.79

True-positive indicates patients with a favorable outcome correctly predicted to have a favorable outcome (DRS of 6 or lower).

## Discussion

This study tested the hypothesis that PPS representation underlies a minimal form of self-consciousness, and that an EEG-based index of PPS representation could thus detect changes in consciousness. In awake healthy participants, high-beta (20–30 Hz) oscillations in a centro-parietal cluster indexed PPS representation, matching typical fronto-parietal PPS regions.^18^ This index persisted during dreaming sleep, when conscious experience was present, but vanished during dreamless sleep, mirroring changes in consciousness state.^47^ In DoC patients, the PPS index correlated with CRS-R scores at admission and robustly predicted clinical outcomes at discharge.

A theoretical link between PPS and minimal selfhood has been proposed and supported by empirical findings.^15^^,^^18^^,^^48^ PPS enables body-environment interactions from a first-person perspective,^13^^,^^49^ and recent studies highlight their relevance during dreaming,^33^ where most interactions occur within PPS.^50^ Our findings provide neurophysiological evidence of multisensory integration during sleep (see van der Heijden et al.^51^ for subjective reports) and link body-centered multisensory responses to conscious experience.

The relationship between PPS representation and dream content appeared unrelated to the incorporation of external stimuli into dreams. Although auditory and tactile stimuli evoked neural responses in both conscious and unconscious periods, participants rarely reported incorporating these stimuli into their dreams, which are predominantly visual. Any incorporation that did occur seemed indirect and inconsistent. Thus, we propose that the PPS index does not reflect the awareness of the experimental stimuli, but rather measures the brain’s capacity to sustain conscious experience, irrespective of whether the probing stimuli are perceived or not. This is reminiscent of methods combining transcranial magnetic stimulation (TMS) and EEG to obtain complexity and integration measures, where the TMS pulse probes consciousness without eliciting a subjective percept.^52^ Our approach complements other strategies for studying the neural correlates of consciousness, including those that examine sensory processing as a proxy for stimuli awareness^53^ or analyze spontaneous brain activity without external stimulation to infer conscious processing.^54^^,^^55^

Our clinical data further support the relationship between PPS and consciousness. In DoC patients, the PPS index correlated with residual consciousness.^29^ Patients with positive PPS indices at admission, resembling healthy wakefulness, showed better outcomes, while negative indices, akin to unconscious sleep, predicted poorer recovery. These results, along with dream report results, suggest that the PPS index can detect covert consciousness, offering complementary insights to CRS-R, which relies solely on observable behaviors.

Interestingly, the PPS index correlated more strongly with the outcome index at patient discharge than with the CRS-R at the time of the recording. This may depend on a generic higher sensitivity of the outcome index, based on extensive clinical assessments at discharge, as compared to the sole CRS-R. More intriguingly, this may relate to intrinsic limitations of the CRS-R scale, only partially predicting the long-term clinical outcome, as exemplified by its limitations in CMD patients.^56^ The CRS-R scale is based purely on the presence of behaviors indicative of consciousness, thus capturing only overt signs of consciousness. In contrast, our PPS index does not require any behavioral response to detect consciousness. By measuring patients’ residual multisensory integration in PPS at the beginning of their rehabilitation, our method captures differences in patients’ preserved consciousness and potential of interaction during recovery, not detected by the CRS-R, and thus may further contribute to the outcome prediction.

Further analyses suggested that, in certain cases, the PPS index may achieve a better PPV and specificity in detecting favorable outcome patients, compared to the CRS-R. This further supports the idea that the PPS index may provide complementary information to clinical evaluation. Importantly, this result should be interpreted in the context of a clinically relevant trade-off between sensitivity and specificity. While maximizing sensitivity is critical to avoid overlooking patients with any potential for recovery, higher PPV and specificity may be valuable when the goal is to tailor and optimize rehabilitation strategies for patients with higher chances of favorable outcome. Moreover, combining the PPS index with the CRS-R further improved classification performance, PPV, and specificity, highlighting the benefit of a multimodal assessment strategy. While these results already underscore the value of integrating behavioral and neural markers, even greater predictive power could potentially be achieved by including additional modalities, such as fMRI-based connectivity measures, which accurately predicted the clinical outcome and showed remarkable agreement with the PPS index. We provided exploratory evidence in this sense, showing that the predictive power of the PPS index improves when it is combined with mesocircuit integrity. Although this finding should be more robustly benchmarked in future studies using larger multimodal datasets, it highlights that our index may yield optimal performance when integrated in a battery of clinical and paraclinical tests.

Importantly, the link between the PPS index and clinical outcome could not be reduced to the response to any specific experimental condition or to any combination of conditions other than the PPS index (see, e.g., Figure 4G). This is in line with our overarching hypothesis that a body-centered, multisensory representation of potential interactions between bodily and external stimuli detects an important component of consciousness. Our results in patients demonstrate that such component of consciousness is also linked to clinical trajectories when recovering from DoCs.

It has been previously demonstrated that supplementing standard clinical evaluations with a detailed assessment of spontaneous motor behavior (MBT-r) can identify a subset of patients exhibiting signs of interaction with their environment, classified as c-CMD.^57^^,^^58^ c-CMD patients show distinct recovery trajectories and significantly better long-term outcomes.^56^^,^^59^^,^^60^ Consistently, these patients exhibited a higher PPS index compared to those with a true DoC. This suggests that quantitative neurophysiological markers, such as the PPS index, can complement existing clinical assessments, which rely on observable behaviors to infer consciousness. Such behavioral assessments have intrinsic limitations^61^^,^^62^ and may misclassify up to 40% of patients.^63^ The PPS index provides an additional, objective measure to identify covert consciousness and improve diagnostic accuracy in distinguishing between c-CMD and true DoC patients.

Furthermore, patients with poorer outcomes showed more severe lesions to the forebrain mesocircuit and reduced DMN-DAN anticorrelation.^42^ The latter is a hallmark of consciousness disruption in deep sleep or anesthesia^45^^,^^64^ and a key mechanism mediating interactions with the environment.^45^ Here, DMN-DAN anticorrelation was associated with a higher PPS index and with preserved forebrain mesocircuit integrity. Importantly, when accounting for shared variance between DMN-DAN coupling and mesocircuit integrity, only the latter still predicted the PPS index. This is compatible with mesocircuit integrity driving both PPS function and DMN-DAN coupling and is possibly explained by the notion that the mesocircuit acts as a global arousal regulator.^65^^,^^66^ This suggests that both the DMN-DAN anticorrelation and the PPS index may constitute two emerging faces of structural and functional integrity of key circuits for consciousness, whose functionality may break down either temporarily, as in deep sleep, or permanently, in DoC patients.

Beta oscillations, here used as a marker of PPS representation, are thought to underlie feedback signals in cortical networks^67^^,^^68^ and have been related to multisensory^69^ and sensorimotor^70^ processing. Recent studies have specifically linked individual differences in beta-band synchronization and PPS representation^71^ and stimulus-induced beta-band desynchronization with the activation of the PPS system.^72^ The role of beta oscillations in sleep and altered states of consciousness is not fully understood. Intracranial recordings have shown that while high-gamma responses to auditory stimuli, reflecting local spiking activity, remain comparable between wakefulness and sleep, beta-band desynchronization occurs only during wakefulness.^73^ This suggests that the combination of feedforward gamma and feedback beta signaling, which characterizes conscious perception, is disrupted during unconscious states. Our findings may reflect a similar mechanism, where the absence of beta feedback signaling specifically affects the space-dependent processing of information underlying PPS representation.

Since this is the first work studying oscillatory features of audiotactile PPS representation, we restricted our analyses to the 20–30 Hz frequency range, conventionally accepted in literature and linked to PPS representation.^71^^,^^72^ However, the significant range in our two cohorts did not entirely overlap with the high-beta range, suggesting the exact frequency range yielding the best predictive power may be slightly different. Future studies could capitalize on these first observations to further refine the frequency definition of our index.

In conclusion, we demonstrated that PPS representation, a marker of the Bodily Self, is linked to consciousness levels in both healthy individuals and DoC patients, despite important differences in the physiology of deep sleep compared to DoC, first of all its reversibility. Understanding the neural mechanisms of PPS could deepen insights into consciousness, its fluctuation during sleep-wake cycles, and its loss in brain injury. PPS representation is plastic,^13^^,^^74^ emerges from statistical regularities during body-environment interactions,^16^ and plays a key role in priming relevant actions.^75^^,^^76^ By connecting PPS representation with consciousness levels and clinical outcome in DoC patients, our findings may help improve early detection of recovery potential and open avenues for therapeutic interventions.

### Limitations of the study

Some limitations have to be acknowledged for this study. Clinical outcomes were assessed only at discharge, with no follow-ups, limiting long-term predictive insights. Recovery trajectories can extend beyond initial evaluations,^77^^,^^78^ and longer follow-ups are warranted. Additionally, further research is needed to pinpoint the precise neural mechanisms and structures underlying our beta desynchronization index of PPS representation and its modulation during physiological and pathological consciousness states.

## Resource availability

### Lead contact

Further information and requests for resources should be directed to and will be fulfilled by the lead contact, Andrea Serino (andrea.serino@unil.ch).

### Materials availability

This study did not generate new unique reagents or materials.

### Data and code availability

• The EEG and questionnaire/clinical data reported in this study cannot be deposited in a public repository due to their sensitive nature. Requests for data access should be addressed to the lead contact (andrea.serino@unil.ch) and should include the applicant’s organization, the intended use of the data, and details regarding data storage. We aim at granting access for research purposes as long as safe data transfer and storage is guaranteed, subject to approval by the Sleep Center (sleep experiment) and NPR Unit (patient data) at Lausanne University Hospital, in accordance with the data-sharing constraints approved by the responsible ethics committee.
• This paper does not report original code.
• Any additional information required to reanalyze the data reported in this work paper is available from the lead contact upon request.

## Acknowledgments

This work was supported by a 10.13039/501100001711Swiss National Science Foundation Professorship grant, grant number 163951, http://www.snf.ch, awarded to A. Serino This work was further supported by the 10.13039/501100001711Swiss National Science Foundation Ambizione grant PZ00P3_173955 and the 10.13039/100000190ERC grant Dreamscape 101039782 awarded to F.S.

## Author contributions

T.B., analyzed the data and wrote the original draft; G.R., analyzed the data and contributed to the original draft; J.J., collected clinical data and reviewed the paper; J.F., conceptualized the study and collected sleep data; B.D. and J.C., collected and preprocessed sleep data; A. Stephan, developed the experimental setup; C.F. and M.L.D.S., collected DoC EEG data; M.G., contributed to preprocessing DoC data; P.P. and V.D., collected and analyzed MRI data and reviewed the paper; M.D.L., reviewed the paper; J.-P.N., conceptualized the study and reviewed the paper; E.M. reviewed the paper and supervised data analysis; K.D., conceptualized the study and reviewed the paper; F.S., conceptualized the study, reviewed the paper, and supervised data analysis; A. Serino, conceptualized the study, wrote the original draft, and supervised data analysis.

## Declaration of interests

The authors declare no competing interests.

## STAR★Methods

### Key resources table

**Table:** 

REAGENT or RESOURCE	SOURCE	IDENTIFIER
**Biological samples**		

Human EEG data (healthy participants)	This study	Not applicable
Human EEG data (DoC patients)	This study	Not applicable

**Critical commercial assays**		

Pittsburgh Sleep Quality Index (PSQI)	Buysse et al.^79^	Not applicable
Insomnia Severity Index (ISI)	Morin et al.^80^	Not applicable
Epworth Sleepiness Scale (ESS)	Johns^81^	Not applicable
Fatigue Severity Scale (FSS)	Krupp et al.^82^	Not applicable
Morningness–Eveningness Questionnaire	Horne & Östberg^83^	Not applicable
Coma Recovery Scale–Revised (CRS-R)	Giacino et al.	Not applicable
Motor Behavior Tool–revised (MBTr)	Pignat et al.^58^	Not applicable

**Deposited data**		

EEG dataset	This study	Available upon request

**Experimental models: Cell lines**		

Homo sapiens	This study	Healthy participants and patients

**Software and algorithms**		

GeoSource	Electricsl Geodesic, Inc	Not applicable
MATLAB	MathWorks	RRID:SCR_001622
EEGLAB toolbox	Delrome & Makeig	RRID:SCR_007292
FieldTrip toolbox	Donders Institute	RRID:SCR_004849
E-Prime 2	Psychology Software Tools	Not applicable

**Other**		

High-density EEG system (256-channel)	Electrical Geodesics, Inc.	EGI system
Low-density EEG system (16-channel)	g.tec medical engineering GmbH	g.USBamp/g.Nautilus
Digitimer DS7AH	Digitimer Ltd.	DS7AH
Functional electrical stimulator	MEDEL Medical Electronics	MOTIONSTIM 8
Loudspeakers (auditory stimuli)	Logitech	Z120

### Experimental model and study participant details

#### Healthy participants - Wakefulness and sleep

Fifteen healthy volunteers (age 22 ± 2 years, range 19–25 years, 8 females) participated in the study and were screened for psychiatric, neurological, and sleep disorders. All volunteers had good sleep quality, as assessed by the Pittsburgh Sleep Quality Index (PSQI score <55^79^) and the Insomnia Severity Index (ISI score <10^80^). None of the participants had an extreme chronotype, as determined using the Morningness–Eveningness Questionnaire,^83^ nor excessive fatigue as assessed by the Fatigue Severity Scale (FSS ≤36^82^) or excessive daytime sleepiness as assessed by the Epworth Sleepiness Scale (ESS <10^81^).

The sample size was determined based on previous studies investigating sleep modulation using external stimulation, in which typically 15 participants were recruited.^84^^,^^85^^,^^86^ The study was approved by the ethical committee of the canton of Vaud, Switzerland (CER-VD 2017-01588, CER-VD 2016-00812), and was conducted in accordance with the Declaration of Helsinki. All participants provided written informed consent. Experiments were performed at the Lausanne University Hospital sleep center.

Due to technical issues with EEG triggers, data recorded during sleep for two participants were not analysable. Hence, analyses on sleep data have been conducted on the remaining 13 participants. We verified that the main PPS index during wakefulness in the high beta range could be replicated in this subset of subjects (see Figure S10). Furthermore, one participant did not present periods of REM sleep, resulting in a final sample of 12 participants for analyses involving REM sleep.

#### Patients with disorders of consciousness

Seventy-two patients with disorders of consciousness (19 females; age = 48.9 ± 17.6 years, range = 19–84 years) participated in this second study, which investigated the audio-tactile PPS paradigm in true DoC and putative cognitive motor dissociation (CMD) patients, following its previous evaluation in healthy participants. Participants provided informed consent to take part in the study, which was approved by the local ethical committee of the canton of Vaud, Switzerland (CER-VD, 142/09). Consciousness levels and diagnoses were determined by clinical neurologists and neuropsychologists using the Coma Recovery Scale–Revised (CRS-R).^37^

Each patient underwent multiple testing sessions (average 2.5, range 1–10) across the DoC spectrum. The number of sessions per patient was influenced by clinical requirements, primarily patient availability for research and the duration of their stay at the acute neurorehabilitation unit of the University Hospital of Lausanne (CHUV), Switzerland. For the present analyses, we analyzed only data from the first session, which was available for the largest cohort of patients.

The experiment was conducted on average 30.7 days post–brain injury (SD = 14.1, range = 6–83 days). At discharge, which occurred on average 24 days after EEG recording (SD = 14.1, range = 1–64 days), patient recovery was assessed by expert clinicians using a standardized evaluation, which included four different clinical scales combined to evaluate potential residual cognition and motor intent. Namely, the Rancho Los Amigos (RLAS),^87^ Early Barthel Index (ERBI),^88^ Functional Ambulation Category (FAC)^89^ and Disability Rating Scale (DRS)^90^ were collected.

Throughout their clinical stay, and prior to admission to the unit, patients were repeatedly evaluated clinically by neurologists and neuropsychologists using the Coma Recovery Scale-Revised (CRS-R) and the Motor Behavior Tool (MBTr^57^). The MBTr is a clinical motor observation tool designed to describe the spontaneous (without stimulation) motor behavior in a non-task related way (akinesia versus reflex motor pattern of decortication and decerebration) and detect subtle non-reflexive movements and ”positive“ responses that suggest intentionality, that are not considered by the CRS-R, along with a systematic search for obstacles limiting their interaction capabilities. Preserved conscious integration is considered present according to this tool when at least one positive item is noted, leading to the classification of the patient as having clinical CMD (c-CMD). The MBTr has demonstrated the ability to identify a subgroup of patients exhibiting motor intention, the presence of which predicts favourable recovery.^56^^,^^57^^,^^91^ Clinical cognitive motor dissociation (c-CMD) is different from classical CMD, as it does not rely on task-based neuroimaging or electrophysiological command-following paradigms. Thus, we refrain from directly comparing detection rates between c-CMD and CMD.

### Method details

#### Healthy participants - Wakefulness and sleep

##### Stimulus types, delivery, and intensity

Stimuli could be auditory (near or far), tactile, or a combination of the two, resulting in three unisensory and two multisensory stimulus conditions: auditory near (AN), auditory far (AF), tactile (T), audio-tactile near (ANT), and audio-tactile far (AFT). Auditory stimulation consisted of 100-ms bursts of white noise generated using E-Prime 2 (Psychology Software Tools) and delivered through one of two commercial loudspeakers placed either 30 cm (near) or 130 cm (far) above the participant’s chest. Tactile stimuli consisted of a 5-ms electrical stimulation delivered to the chest, 2 cm below the manubrium sternum, using a Digitimer DS7AH stimulator. Multisensory stimuli were delivered such that the onset of auditory and tactile stimulation was synchronized.

The intensity of stimuli had to be carefully adjusted in order to obtain measurable responses without awakening participants in the sleep session. For auditory stimuli, we started from determining the auditory threshold. The minimal volume necessary for a sound to be perceived was determined for each individual using an up-down staircase procedure. During this task, participants were lying in bed with eyes closed and were instructed to detect sounds of different intensities. The experiment involved three blocks of 30 trials each. At the beginning of each block, the sound level was set at a value above threshold for all participants (46 dB). The sound level was decreased by 2 dB following a positive response (e.g., “I hear a sound”) and increased by 2 dB following a negative response (e.g., “I do not hear anything”). Volumes presented during the last ten trials of each block were averaged, and a grand average across the three blocks was computed to estimate the auditory threshold. Finally, the stimulus intensity was defined as the individual auditory threshold plus 40% (mean 51 dB, SD = 4.01). Intensity was adjusted for both near and far sounds so that the sound pressure at the participant’s ear was the same.

The intensity of tactile stimuli was determined based on participants’ subjective perception of an intensity comparable to the PPS auditory stimulation. Participants lay in bed with eyes closed while the PPS auditory stimulus was delivered, followed immediately by a tactile stimulation. The electrical stimulation consisted of a single, constant-voltage, rectangular monophasic pulse. Participants indicated whether the tactile stimulation needed to be “more intense” or “less intense” to match the perceived intensity of the auditory stimulus. The task began with a tactile stimulation above threshold for all participants (5 mA). Tactile intensity was decreased by 0.5 mA intensity when the subject’s response was positive (e.g., “The tactile intensity should be higher to match the sound intensity to reach the same intensity as the sound”) and increased by 0.5 mA when the response was negative (e.g., “I would like less tactile intensity to reach the same intensity as the sound”). The PPS auditory stimulus was administered between each tactile stimulation. The task ended once participants reported that the tactile stimulation matched the auditory stimulus in perceived intensity. This value was choosen as the tactile stimulation level for the PPS experiment (mean 8.7 mA, SD = 5.5 mA). The values obtained with this procedure were in the same range as those employed in previous PPS studies.

##### Experimental protocol

Before the sleep session, participants were lying in bed with their eyes closed and completed three blocks of PPS stimulation while EEG was recorded. Each block consisted of 110 trials, including 22 unisensory tactile stimuli (T), 22 unisensory auditory stimuli presented at a near distance (AN), 22 unisensory auditory stimuli presented at a far distance (AF), 22 audio-tactile stimuli presented at a near distance (ANT), and 22 audio-tactile stimuli presented at a far distance (AFT). The order of trials was fully randomized within each block. The inter-trial interval ranged between 2.5 and 3 s, jittered in five random steps of 100 ms. Each block lasted approximately 7 min. No behavioral response was required from participants during the task.

After completion of the awake session, participants slept from approximately 11:30 p.m. to 6:30 a.m. while EEG data were continuously recorded. During the night, 5-min blocks of stimulation were alternated with 5-min blocks without stimulation. Stimulation blocks included the same five randomized stimulus conditions used in the awake PPS session: unisensory tactile, unisensory auditory near, unisensory auditory far, audio-tactile near, and audio-tactile far. Stimulation blocks were administered during stable sleep stages (N2, N3, or REM; N1 was avoided). The order of stimulation and no-stimulation blocks was randomized and administered independently of sleep stage. Across the night, participants were awakened on average nine times (SD = 1.7) and asked to complete a consciousness questionnaire assessing the presence and content of conscious experience. Awakenings could occur following either stimulation or no-stimulation blocks in randomized order.

##### Consciousness questionnaire

During the night, participants were awakened by a computerized alarm sound lasting 1.5 s, following which they were instructed via interphone to answer a questionnaire designed to assess sleep-related conscious experiences.^32^ After ten seconds from the alarm, participants were first asked to report what had been going through their mind a moment before awakening and to indicate whether they had a conscious experience and could remember its content (dreaming experience, DE), a conscious experience without recallable content (dreaming experience without recall, DEWR), or no conscious experience (NE). In cases participants reported a dreaming experience and could recall the content (DE), they were asked to rate, on an integer scale from 1 to 5, the extent to which their experience included sensory components (e.g., tactile, auditory, visual) and the degree to which these sensory experiences were integrated into their dream. Participants were also asked whether the content of their dream was related to the experimental stimuli (yes/no) and were invited to provide a free verbal description of the content of their dream. Following DEWR or NE reports, they were directly asked to rate how awake or asleep they felt immediately before the alarm, using a scale from 1 to 5.

##### EEG recording and preprocessing

EEG data were recorded using a high-density EEG system (256 channels; Electrical Geodesics, Inc., Eugene, Oregon) with a sampling rate of 500 Hz and Cz as a reference electrode. Four electrodes positioned near the eyes were used to monitor eye movements, and electrodes placed over the masseter muscles were used to record muscle tone.

The EEG signal was band-pass filtered offline between 0.5 and 45 Hz, and a notch filter at 50 Hz was applied to remove line noise. Sleep scoring was performed on 30-s epochs according to standard criteria.^92^ Channels containing artifactual activity were visually identified and replaced by interpolation over neighboring channels using spherical splines (NetStation, Electrical Geodesic). Ocular, muscular, and cardiac artifacts were removed using Independent Component Analysis (ICA) implemented in EEGLAB.^93^ ICA components exhibiting activity patterns and scalp topographies characteristic of artifactual sources were identified through visual inspection and removed.^94^ Epochs containing awakenings during the sleep session were excluded from subsequent EEG analyses. ICA computation and component rejection were performed separately for each analyzed sleep stage (N2, N3, and REM).

Stimulus-locked epochs ranging from −1 s to +2 s relative to stimulus onset were extracted from the continuous EEG data. Each epoch was baseline-corrected by subtracting the mean signal value computed over the baseline period for each electrode. All subsequent EEG analyses were performed on average-referenced data. Preprocessing of EEG data from the wake and sleep session followed the same procedure, except for sleep scoring.

##### Cortical sources Reconstructuon

To localize the PPS effect at the cortical level, source localization was performed on the preprocessed EEG signals using GeoSource software (Electrical Geodesics, Inc., Eugene, Oregon). The forward model was constructed based on individualized electrode geocoordinates, and the source space was constrained to 2447 cortical voxels with a spatial resolution of 7 mm. The inverse solution was computed using standardized low-resolution brain electromagnetic tomography (sLORETA^95^), combined with Tikhonov regularization (λ = 10^−2^) to account for variations in signal-to-noise ratio. The software provided three-dimensional (xyz) temporal evolutions for each cortical voxel. MATLAB was used to reconstruct a one-dimensional time series per voxel by projecting the xyz activity onto the voxel’s principal orientation using singular value decomposition (SVD).

#### Patients with disorders of consciousness

##### Stimulus types, delivery, and intensity

Auditory stimuli were presented at near and far distances and consisted of 50 ms bursts of white noise delivered through loudspeakers (Z120 Portable Speakers, Logitech, Lausanne, Switzerland) positioned at 5 cm and 75 cm from the participant’s extended arm in the depth dimension (65.2 dB SPL and 64.1 dB SPL, respectively). Tactile stimuli were delivered to the participant’s arm for 50 ms at a frequency of 35 Hz using functional electrical stimulation (FES; MEDEL Medical Electronics, MOTIONSTIM 8, Innsbruck, Austria). Two electrodes (positive and negative, Flextrode Plus) were placed on the extensor digitorum communis on the dorsal aspect of the arm near the elbow. Stimulation intensity was set at 70% of each participant’s motor threshold, determined immediately before the experiment and ranging from 5 mA to 11 mA. A third electrode placed on the right shoulder served as an earth ground to reduce electrical artifacts from FES stimulation. In multisensory trials, auditory and tactile stimuli were administered synchronously.

##### Experimental protocol

During the experiment, patients were positioned supine at an angle of approximately 130° within a controlled environment with regulated lighting and sound. Each block of audio-tactile stimulation lasted 10 min and comprised 250 trials, including 50 unisensory tactile stimuli, 50 unisensory auditory stimuli presented at a near distance, 50 unisensory auditory stimuli presented at a far distance, 50 audio-tactile stimuli at a near distance, and 50 audio-tactile stimuli at a far distance. The sequence of these stimuli was fully randomized within each block, with an inter-trial interval uniformly distributed between 1.5 and 2 s. Due to clinical considerations, the number of blocks recorded per patient varied, averaging 2.8 blocks per session and ranging from 1 to 3 blocks per session. Brief breaks were provided between blocks.

##### EEG recording and preprocessing

Patients’ EEG data were collected using a 16-channel EEG system (g.USBamp and g.Nautilus, g.tec medical engineering GmbH, Graz, Austria) with a sampling rate of 500/512 Hz and referenced to the right earlobe. Electrodes were positioned to cover motor and somatosensory areas (Fz, FC3, FC1, FCz, FC2, FC4, C3, C1, Cz, C2, C4, CP3, CP1, CPz, CP2, and CP4).

Due to the large number of patients, and with the long-term aim of developing a fully objective clinical assessment, EEG preprocessing was based on an automated procedure for DoC phenotype patients. EEG signals were band-pass filtered offline between 0.5 and 40 Hz, with an additional notch filter to eliminate power line noise. Epochs were extracted from continuous EEG data spanning −100 ms–500 ms relative to stimulus onset and were baseline-corrected to the 100 ms pre stimulus onset. Bad channels were excluded based on an amplitude threshold of ±100 μV, and bad epochs were rejected based on automated joint probability measures implemented in EEGLAB. Then, within each session, blocks of epochs were concatenated prior to performing Independent Component Analysis (ICA). Components with less than a 60% likelihood of representing neural activity were excluded from further analysis through EEGLAB’s automated component rejection tool. Following this pre-processing procedure, six patients were excluded due to excessive noise and artifacts that compromised the EEG signals.

### Quantification and statistical analysis

#### Spectral analysis and PPS index definition

In both experiments, spectral analysis was used as the initial processing step and served to derive an index of PPS representation (PPS index) from oscillatory EEG responses.

##### Spectral analysis – Experiment 1

For each epoch, the Power Spectral Density (PSD) was computed using Welch’s periodogram method with a Hamming window of 0.5 s, 50% overlap between segments, and a frequency resolution of 1 Hz. PSDs were calculated separately for the baseline and post-stimulus periods. PSDs were then averaged across trials for each stimulus condition. Post-stimulus PSDs were normalized relative to an average baseline PSD, computed as the mean PSD across all five conditions. This procedure resulted in a normalized post-stimulus PSD for each subject and stimulus condition (tactile, auditory near, auditory far, auditory near + tactile, and auditory far + tactile). Normalized post-stimulus PSDs were subsequently averaged within predefined frequency bands: theta (4–8 Hz), alpha (8–12 Hz), low beta (12–20 Hz), high beta (20–30 Hz), and gamma (30–40 Hz).

##### Spectral analysis – Experiment 2

For each epoch, the Power Spectral Density (PSD) was calculated for post-stimulus using Welch’s periodogram method with a Hamming window of 0.25 s, 50% overlap between segments, and a frequency resolution of 1 Hz. Subsequently, the PSDs were averaged across trials for each stimulus condition. The post-stimulus PSDs were then normalized relative to an average baseline PSD, computed as the mean PSD across all five conditions. This procedure resulted in a normalized post-stimulus PSD for each subject and stimulus condition (tactile, auditory near, auditory far, auditory near + tactile, and auditory far + tactile). Subsequently, after demonstrating that the average PSD across the 16 channels exhibits a PPS effect within the high beta frequency band identified in the previous dataset, we focused exclusively on computing the spectral analysis in the high beta band. All subsequent statistical analyses of correlation with clinical scales were based on Pearson correlations.

##### PPS index definition

Most previous electrophysiological studies^29^^,^^30^ on PPS representation analyzed signals in the time domain, comparing event related potentials (ERPs) or global field power (GFP) across different stimulation conditions. Here, we applied the same approach to frequency domain features of the response to stimuli, which also show modulations related to PPS processing.^71^^,^^72^^,^^96^ Indeed, transient evoked activity reflected by ERPs is known to change broadly in amplitude, latency and topography between wakefulness and sleep^97^^,^^98^ or in disorders of consciousness.^99^^,^^100^^,^^101^ Thus, to derive a PPS-based marker that can be reliably compared across conscious states, here we developed an EEG measure of PPS representation based on the oscillatory correlates of stimulus processing. Similarly to what was done in previous electrophysiology studies,^28^^,^^29^ the PPS index was defined based on the key functional property of PPS representation, that is, a multisensory and body centered representation linking external stimuli (in this case, auditory) and body-related stimuli (tactile). In terms of comparisons between experimental conditions, this translates to searching for electrodes demonstrating a stronger near-far difference for multisensory stimuli than for unisensory ones, leading to the formula

PPS index = (AN-AF) - (ANT-AFT)

This analysis was performed on the normalized power averaged on the 1 s window following each stimulus for each frequency band. For convenience, since lower power in the key high beta band typically indicates higher neural activation, we reversed the sign of the formula with respect to studies focusing on evoked potentials, where higher amplitude corresponds to higher activation. This way, the PPS index is expected to be positive for high beta power.

#### Healthy participants - Wakefulness and sleep

##### Cluster-based statistics

To assess the statistical significance of differences in Power Spectral Densities (PSDs) while correcting for multiple comparisons across channels, we employed a cluster-based permutation test^102^ focusing on predefined frequency bands. This analysis was conducted using a custom MATLAB script integrating functions from the FieldTrip toolbox. We specifically analyzed 187 internal electrodes, excluding more peripheral ones such as those on the cheeks. The Monte Carlo method was employed to determine statistical significance, involving 5000 random permutations of observed values between two conditions. Specifically, we applied a two-tailed permutation-based *t* test for dependent samples (alpha = 0.05) and implemented cluster-based correction on the spatial dimension. The test statistic was evaluated using the maximum sum under the permutation distribution. To account for spatial adjacency between channels, we computed a neighbor structure for the internal electrodes. Clusters were defined as groups of at least four significant neighboring channels to ensure robustness in spatial clustering. All *p*-values from channel-level analyses are cluster-based corrected as described here.

##### Source level statistics

To evaluate significant voxel activations in the high-beta PPS index, a two-tailed permutation-based *t* test for dependent samples against zero was conducted, utilizing 5,000 random permutations and setting a threshold at *p* < 0.05 (uncorrected) for displaying the results.

##### PPS index-consciousness correlation

These analyses aimed at establishing a link between PPS index and reported consciousness state (NE, DEWR, DE). The analyses were performed on awakenings following PPS stimulation blocks and restrained to NREM sleep due to the limited number of trials in NREM sleep. Similarly to what was done in a previous work on the neural correlates of dreaming, we analyzed responses to stimuli occurring in the 20 s preceding each awakening. Not all subjects presented at least one awakening for each of the three possible consciousness reports, nor a full set of the four experimental conditions needed to compute the PPS index. Thus, we could not simply average PPS indexes across subjects and consciousness reports and perform regular parametric statistics. To overcome this, we used non-parametric statistics based on bootstrapping to estimate the variability of our data and compare consciousness reports. We randomly resampled (with replacement) 13 participants from our original data and computed the PPS index by pooling all trials from all subjects for each consciousness report. The values obtained through this procedure should mimic the distribution of hypothetical replications of the experiment, allowing to estimate confidence intervals on PPS index values and *p*-values.^103^ Confidence intervals (shown in Figure 3E) were estimated as the central 66% of the distribution of bootstrapped PPS index values. P-values were computed by measuring the proportion of resamples yielding larger (or smaller) values in condition A than in condition B. In total, 62 trials in the NE condition, 37 trials in the DEWR condition, and 104 trials in the DE condition were analyzed.

##### Topography of PPS index-consciousness correlation

To investigate how the PPS index interacts with the state of consciousness during sleep, we employed linear mixed models (LMMs). Our objective was to determine whether variations in stimulus distance (near vs. far) and modality (unisensory vs. multisensory), along with the presence (DE) or absence (NE) of dreaming, influence EEG power before the awakenings. For this analysis, the power in beta-high frequency band (20–30 Hz) was considered, since the PPS effect was previously observed in this range during wakefulness. To analyze the three-way interaction, we applied linear mixed models separately for each channel using the following formulation: *power ∼ distance ∗ modality ∗ dreaming + (1|subject)*. In this model, *distance∗modality* captures the PPS effect, while the term *dreaming* indicates the presence (DE) or absence (NE) of dreaming. The model included random intercepts for each subject to account for individual variability in beta-high power.

#### Patients with disorders of consciousness

##### Outcome index

To derive a synthetic index of clinical recovery, we combined different clinical scales at discharge, using a similar approach to Pozeg et al*.*^38^ The index was simply obtained by submitting ERBI, RLAS and DRS scores to a PCA (with scaling and centering), and taking the first component (explaining 93% of variance) as the outcome index.

##### MRI indexes

Methods used to derive the MRI indexes described in Figure 4 are presented in detail in.^38^ Diffusion weighted imaging (DWI) data was denoised, preprocessed, and used to derive fractional anisotropy (FA) maps as described previously in.^104^ We assessed the structural connectivity of the forebrain mesocircuit using the multi-scale probabilistic atlas of the human connectome.^105^ To calculate the mesocircuit structural connectome, we extracted the mean fractional anisotropy (FA) values from the voxels within the white matter bundles that connect the bilateral regions of the forebrain mesocircuit, including the frontal cortex, precuneus, cingulate cortex, thalamic nuclei, and basal ganglia. The biomarker for structural connectivity (mesocircuit FA) was then derived by averaging the FA values across the entire mesocircuit connectome.

To determine the DMN-DAN correlation we first performed an independent component analysis (ICA) to identify these brain networks. Anatomical and resting state fMRI data were preprocessed through the default fMRIPrep pipeline (21.0.2).^106^ To compute resting-state functional connectivity we used data-driven, group ICA^107^ to decompose the preprocessed and smoothed data in 20 spatially ICs. We selected the ICs into the components corresponding to resting state networks, discarding noise components through visual inspection and comparison to the resting state networks templates.^108^ The mean group spatial t-value maps of the remaining ICs were thresholded at t > 4 and used as brain masks for further analyses. The DMN-DAN correlation was then defined as the Pearson correlation of the signal time course in the corresponding masks.

##### Statistical analysis and clinical correlations

Associations between the PPS index in the high beta band and clinical measures were assessed using Pearson correlation coefficients, relating the averaged high beta PPS index to clinical scores collected at the time of EEG recording and to outcome measures at discharge.

## Supplemental information

Document S1. Figures S1–S9 and Table S1

Document S2. Article plus supplemental information
