# Supplementary material for: Multisensory integration in peripersonal space indexes consciousness states in sleep and disorders of consciousness
Source: Cell Rep Med. 2026 Mar 31;7(4):102705. doi: 10.1016/j.xcrm.2026.102705 (PMC13130652; doi:10.1016/j.xcrm.2026.102705)
Supplement: Document S1. Figures S1–S9 and Table S1 [file mmc1.pdf]

Cell Reports Medicine, Volume *7*

Supplemental information

Multisensory integration in peripersonal space indexes consciousness states in sleep and disorders of consciousness

Tommaso Bertoni, Giulia Ricci, Jane Jöhr, Brunella Donno, Jacinthe Cataldi, Julia Fellrath, Aurelie Stephan, Carolina Foglia, Sandro Lecci, Floriane Dauvin, Marina Lopes Da Silva, Mattia Galigani, Polona Pozeg, Vincent Dunet, Marzia De Lucia, Jean-Paul Noel, Elisa Magosso, Karin Diserens, Francesca Siclari, and Andrea Serino

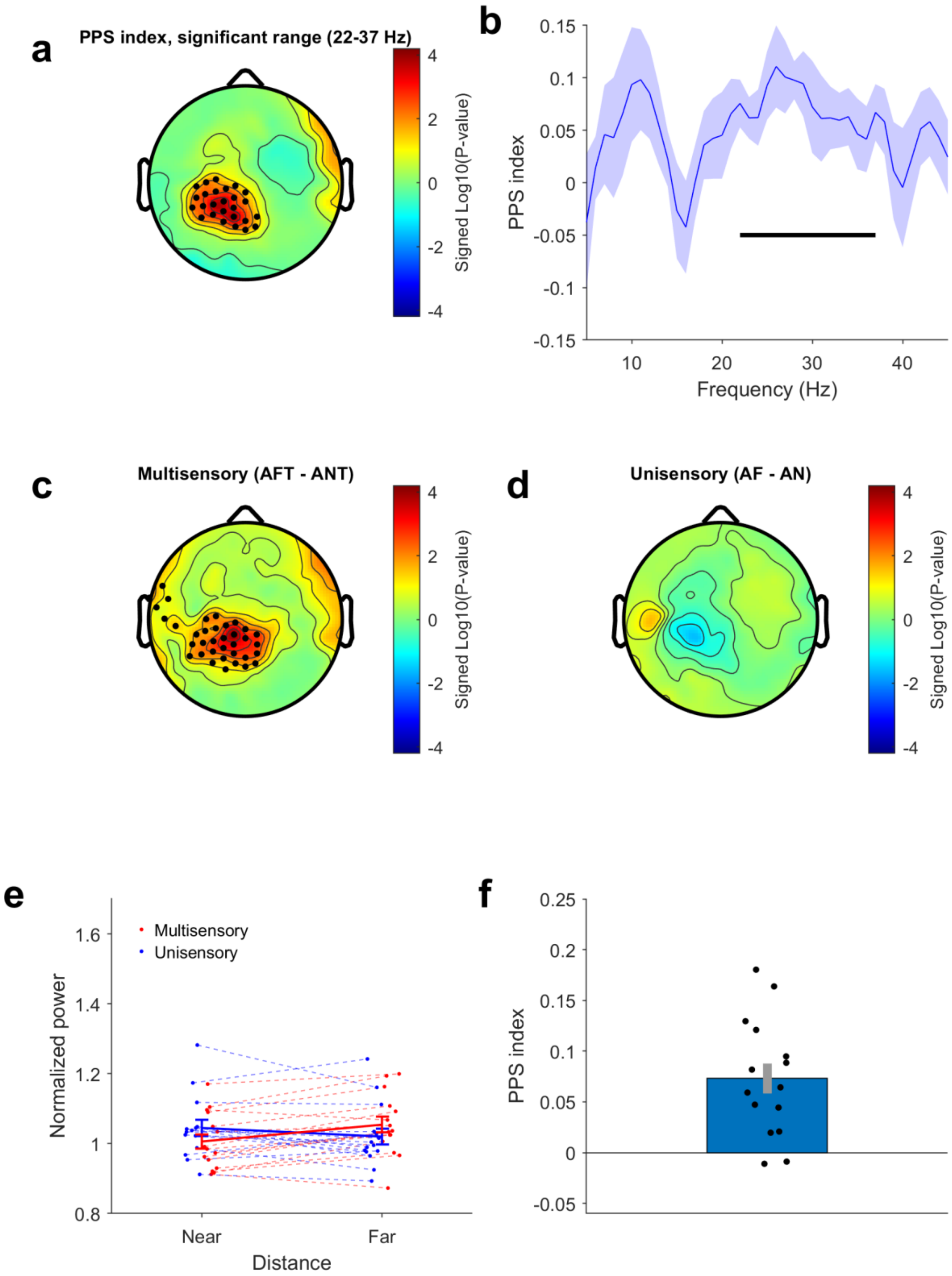

Figure S1. Same as Figure 2 of the main text, with analyses performed on the significant frequency range identified in the centro-parietal cluster (22-37 Hz), related to figure 2. (a) Topography of the PPS index in the 22-37 Hz frequency range. Black dots in the centro-parietal area denote electrodes belonging to the significant cluster. (b) PPS index by

frequency in the significant cluster. Shades indicate standard errors, and the horizontal line indicates frequencies where the PPS index is significantly greater than zero. (c) Comparison between near and far multisensory stimuli (audiotactile far AFT- audiotactile near ANT). Black dots denote electrodes belonging to the significant cluster. (d) Topography of the near-far comparison within unisensory stimuli (audio far AF- audio near AN). (e) Power in the 22-37 Hz band within the cluster identified in (a) in the four experimental conditions for each participant. Thicker lines represent means and error bars standard errors. (f) PPS index in the 22-37 Hz cluster for each participant (the error bar represents the standard error).

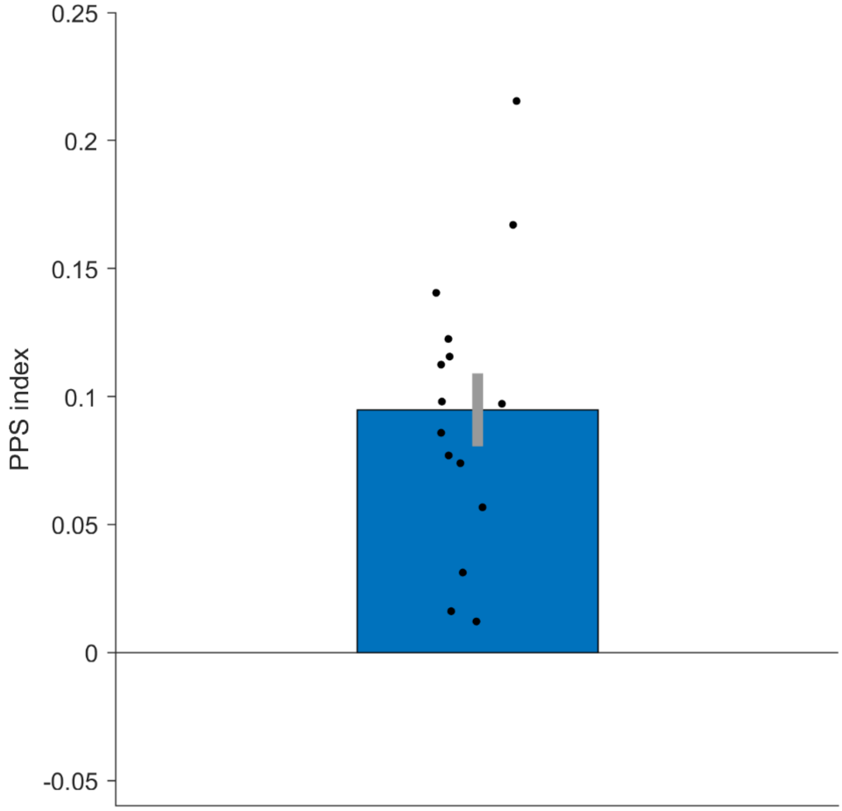

Figure S2. PPS index in the wakeful healthy subjects cohort, restricting the analysis to the 25-30 Hz frequency range, related to figure 2.

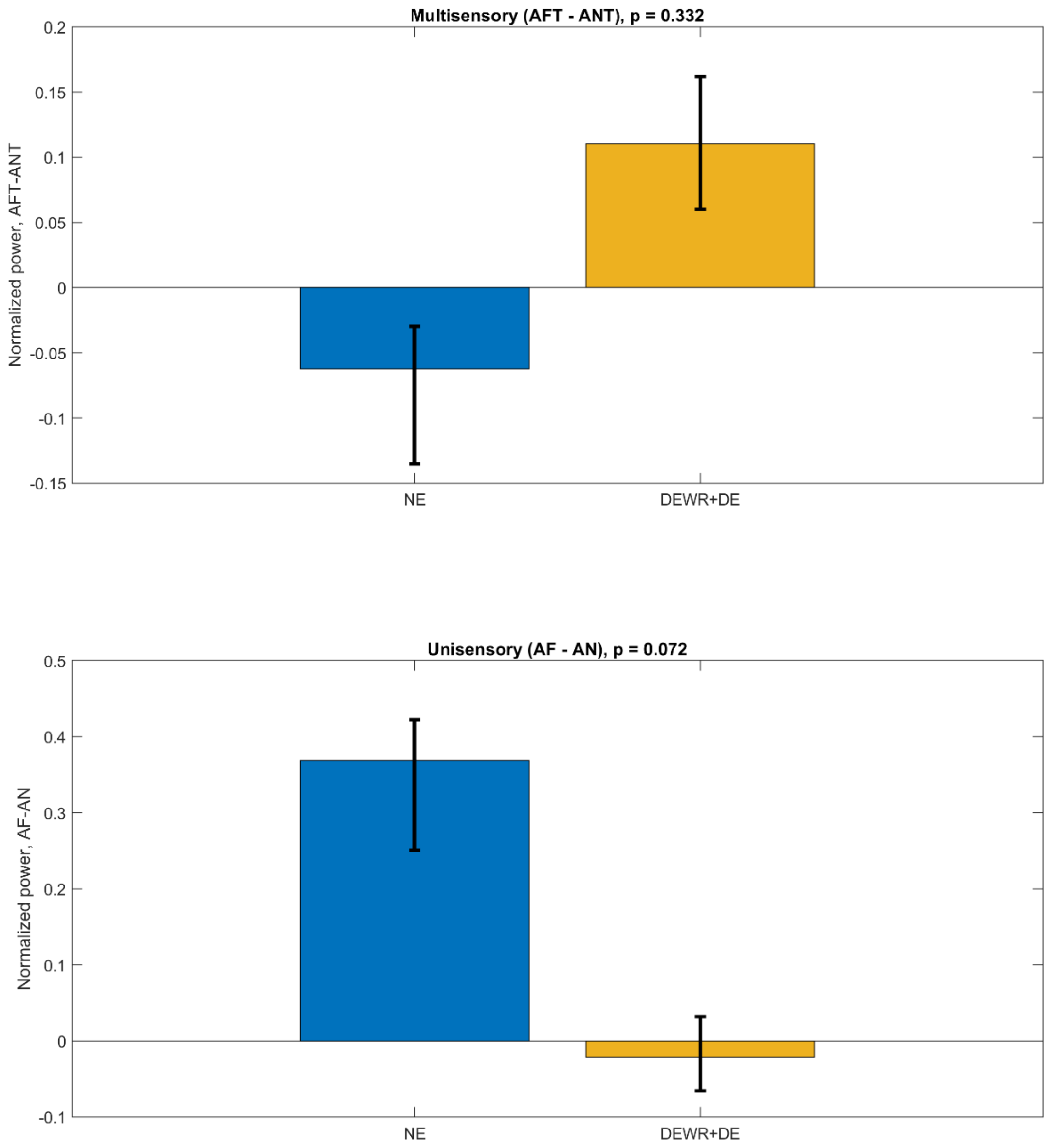

Figure S3. Near-far difference in normalized power for multisensory (top) and unisensory (bottom) trials, compared between unconscious (NE) and conscious (DEWR+DE) sleeping conditions. The p-value refers to the conscious-unconscious comparison, related to figure 3.

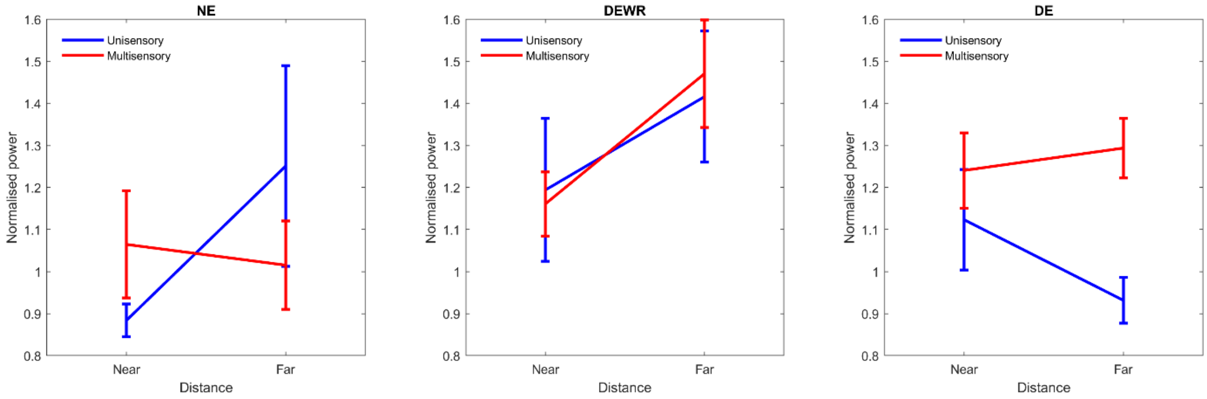

Figure S4. Comparison of normalised high-beta power between consciousness levels showing all the four experimental conditions contributing to the PPS index, related to figure 3.

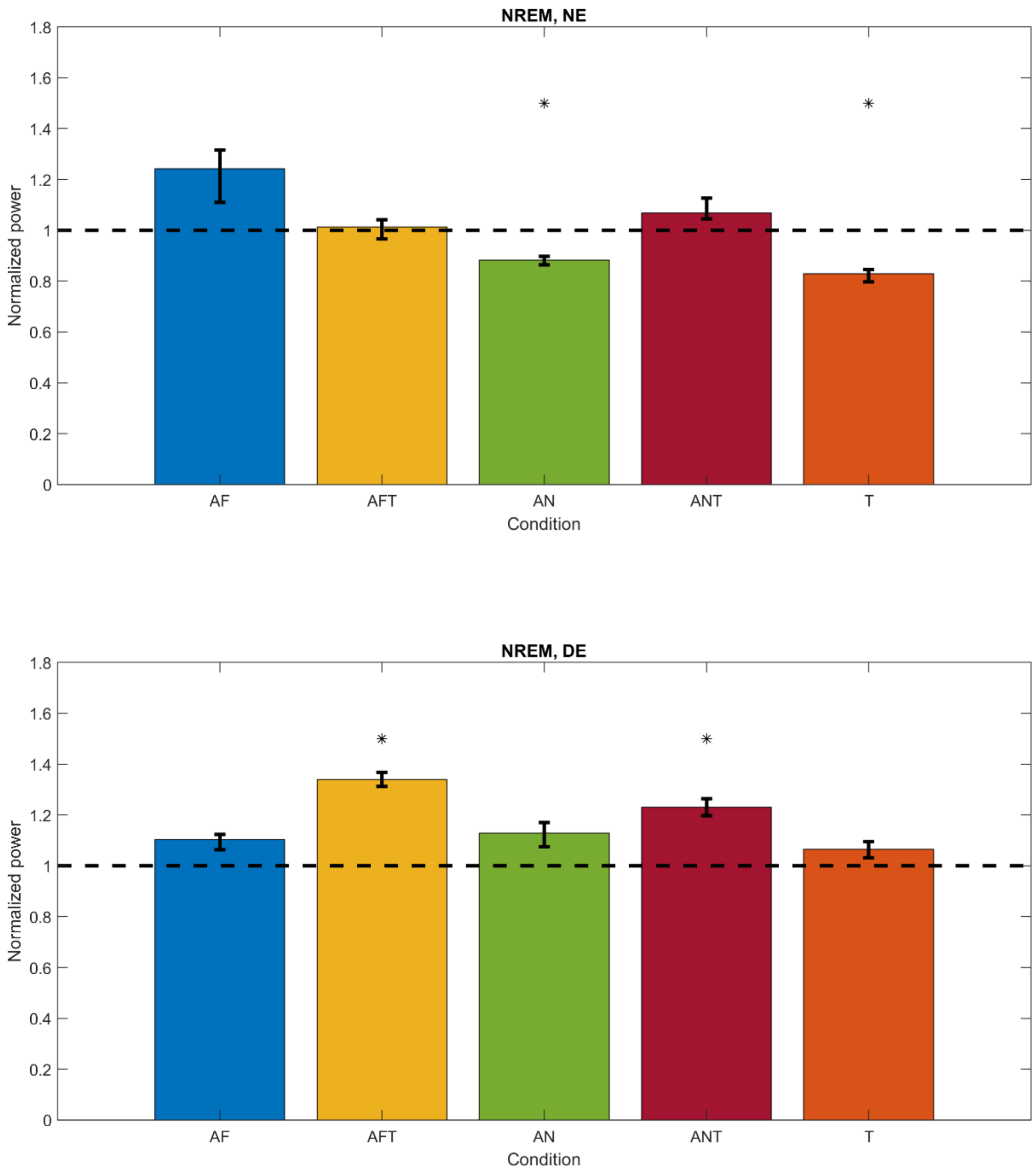

Figure S5. Comparison between stimulus induced response and baseline for the five experimental conditions, in NE (top) and DE (bottom) trials, related to figure 3. Asterisks denote conditions showing a significant difference from baseline (the 1 second pre-stimulus window used for normalization).

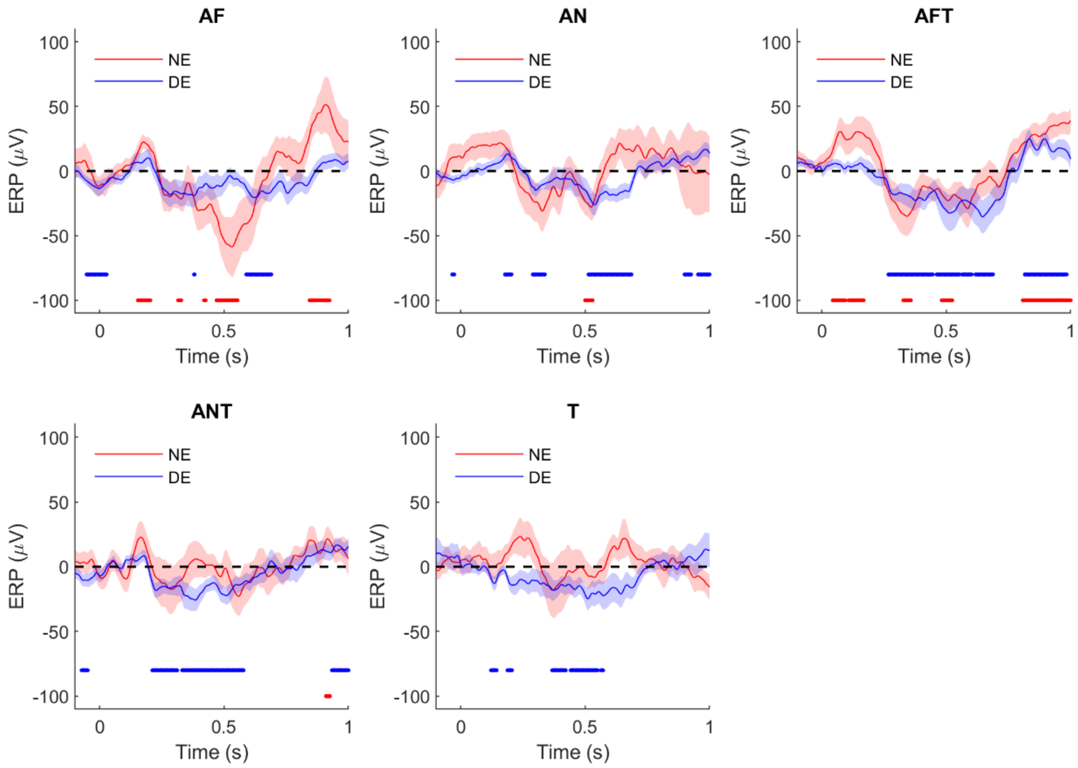

Figure S6. Comparison between stimulus induced response and baseline for the five experimental conditions, in NE (red) and DE (blue) trials, related to figure 3. Dots below the plots denote timepoints showing a significant difference from 0 for NE.

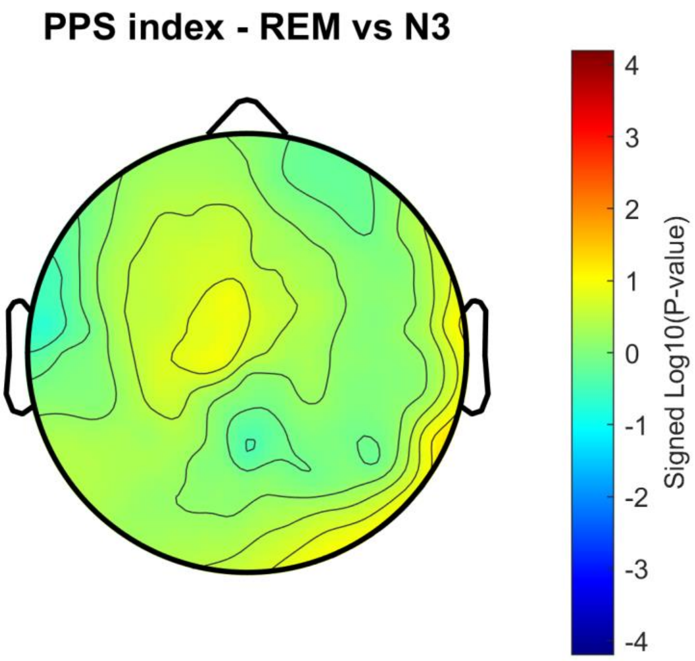

Figure S7: contrast between REM and N3 PPS index. Positive values indicate a higher PPS index for REM compared to N3, related to figure 3.

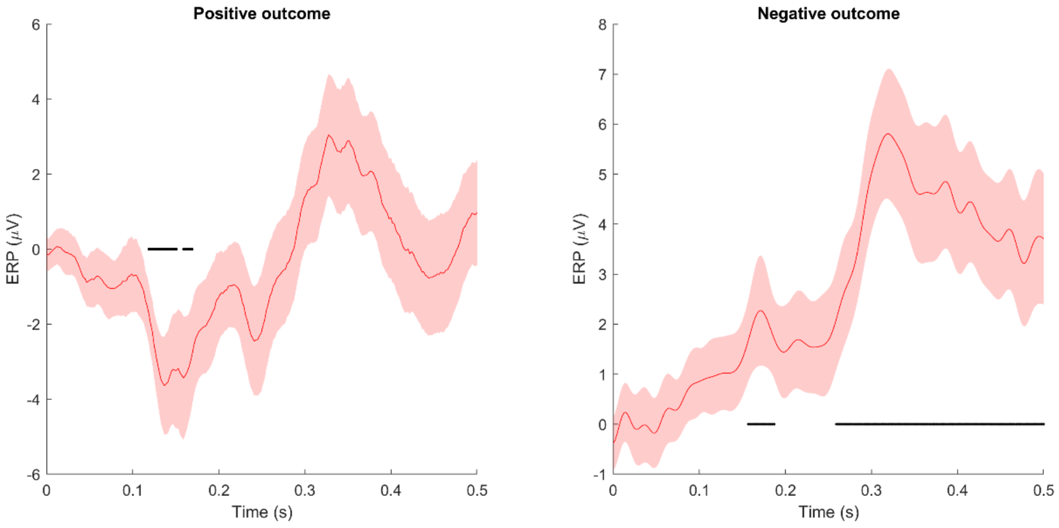

Figure S8. Example of response (sum of AN, ANT, AF, AFT) from the C3 channel in positive and negative outcome patients (based on a median split), related to figure 4. Shades denote standard errors, black lines indicate significant timepoints compared to the pre-stimulus baseline.

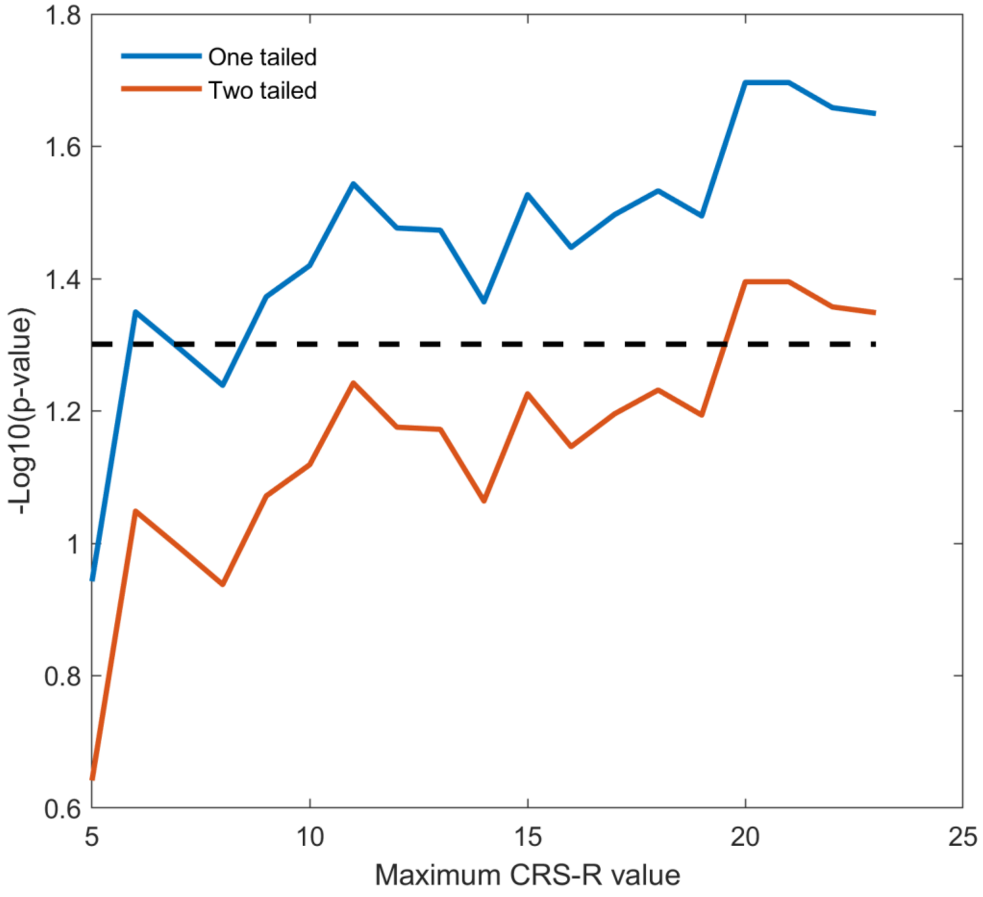

Figure S9. P-values for the coefficient of the PPS index in predicting the outcome index, after setting different maximum thresholds on the CRS-R, to rule out potential ceiling effects on the CRS-R, related to figure 4. The dashed line indicates the conventional significance threshold p=0.05.

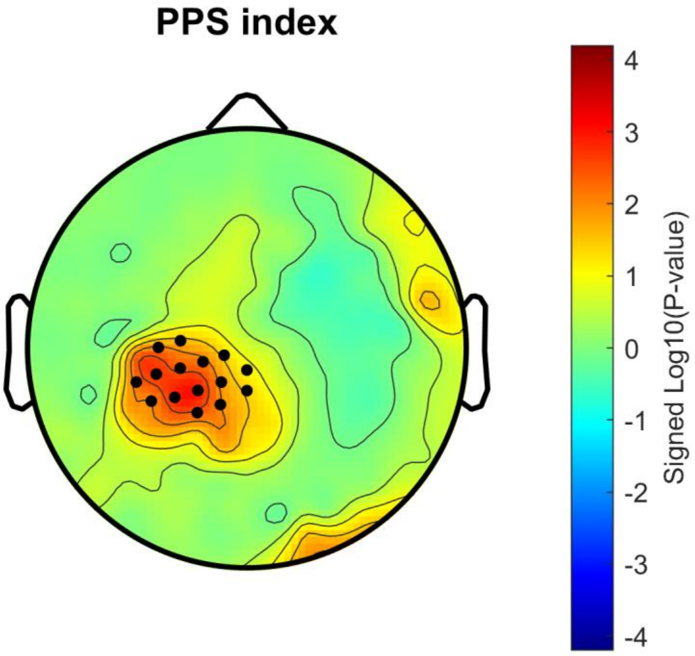

Figure S10: PPS index as in figure 2a, but computed only for the subset of 13 participants with analysable sleep data, related to STAR Methods.

Age range	Sex ass. at birth	Etiology	CRS-R class. at admission	DX based on MBTr	CRS-R at recording	CRS-R class. at recording	Days injury to EEG	Days EEG to disc.	DRS at disc.	ERBI at disc.	RLAS at disc.	Outcome Index	PPS index
70-74	f	Stroke	UWS/VS	cCMD	4	UWS/VS	20	7	24	-225	2	-2.86	-0.37
20-24	m	TBI	UWS/VS	cCMD	23	EMCS	41	3	8.5	15	7	0.91	0.17
60-64	m	Stroke	Coma	cCMD	2	UWS/VS	24	3	29	-225	1	-3.51	-0.08
30-34	m	TBI	MCS-	cCMD	11	MCS-	27	29	4	65	7	1.51	0.66
35-39	m	Anoxia	Coma	DoC	1	Coma	6	1	29	-325	1	-4.03	-0.27
55-59	m	Stroke	UWS/VS	cCMD	3	UWS/VS	28	30	11	15	6	0.45	0.14
30-34	f	Stroke	UWS/VS	cCMD	7	MCS-	9	48	20	-275	4	-2.27	0.03
25-29	m	TBI	Coma	DoC	2	Coma	11	25	29	-275	1	-3.77	-0.67
70-75	m	TBI	UWS/VS	cCMD	9	MCS-	38	50	11.5	-20	6	0.23	-0.15
65-69	m	COVID-19 enceph-a-lopathy	UWS/VS	cCMD	23	EMCS	44	14	5	0	10	1.9	0.21
65-69	m	Stroke	UWS/VS	cCMD	23	EMCS	26	49	6	30	7	1.18	0.35
60-64	m	TBI	MCS-	cCMD	13	MCS-	40	30	17	-20	5	-0.47	-0.02
45-49	f	Stroke	MCS-	cCMD	13	MCS+	29	14	12	-70	5	-0.34	-0.36
45-49	m	TBI	MCS-	cCMD	23	EMCS	33	16	5	10	8	1.42	-0.05
45-49	f	Stroke	MCS-	cCMD	23	EMCS	55	7	4.5	65	9	2	0.08
70-74	m	Anoxia	MCS-	cCMD	6	UWS/VS	31	21	15	-65	5	-0.54	-0.3
40-44	m	TBI	Coma	cCMD	8	MCS-	13	34	7.5	-20	6	0.54	0.07
20-24	f	TBI	UWS/VS	cCMD	23	EMCS	12	11	4	85	9	2.15	0.26
35-39	m	Anoxia	UWS/VS	DoC	5	UWS/VS	37	31	29	-325	1	-4.03	-0.41
55-59	m	Stroke	MCS-	cCMD	23	EMCS	20	14	4.5	20	8	1.51	0.02
25-29	f	TBI	UWS/VS	cCMD	7	UWS/VS	24	21	11	30	6	0.52	0.17
75-79	f	Stroke	UWS/VS	cCMD	16	MCS+	21	32	12.5	-20	5	-0.12	-0.03
45-49	m	Anoxia	Coma	cCMD	15	MCS+	22	25	4	70	8	1.8	0.06
55-59	m	HC	Coma	DoC	3	Coma	53	28	29	-225	1	-3.51	-0.11
21-25	f	Encephalopathy	MCS+	cCMD	23	EMCS	131	24	5.5	25	7	1.21	0.13

15-19	f	TBI	UWS/VS	cCMD	7	MCS-	33	64	9.5	-70	6	0.12	-0.07
70-74	m	TBI	Coma	cCMD	11	MCS-	36	26	5	30	7	1.25	-0.31
30-34	m	TBI	UWS/VS	cCMD	22	EMCS	28	6	3	90	9	2.25	0.75
55-59	m	TBI	UWS/VS	cCMD	14	MCS+	25	22	10	-75	6	0.06	0.09
40-44	m	TBI	UWS/VS	cCMD	14	MCS-	25	35	10	-85	6	0.01	-0.38
50-54	f	Stroke	Coma	cCMD	10	MCS-	45	30	13	-115	5	-0.64	0.13
50-54	m	Anoxia	UWS/VS	cCMD	19	EMCS	29	8	5	50	7	1.36	-0.02
30-34	m	TBI	Coma	cCMD	3	Coma	29	44	20	-170	4	-1.73	0.2
80-84	m	TBI	MCS-	cCMD	5	UWS/VS	30	4	18	-170	4	-1.58	-0.21
20-24	m	TBI	MCS+	cCMD	21	MCS+	26	13	4	75	7	1.56	-0.16
20-24	f	Anoxia	MCS-	cCMD	21	EMCS	29	23	5	70	7	1.46	0.32
75-79	m	Anoxia	UWS/VS-	cCMD	13	MCS-	39	35	6.5	-55	6	0.43	-0.36
35-39	m	TBI	UWS/VS	cCMD	23	EMCS	49	6	7.5	-40	6	0.43	0.07
40-44	f	Stroke	UWS/VS	cCMD	21	EMCS	38	18	7.5	-10	7	0.85	0.19
55-59	m	Stroke	Coma	cCMD	6	UWS/VS	40	49	29	-225	1	-3.51	0.01
55-59	m	Anoxia	MCS-	cCMD	14	MCS+	25	10	2	95	7	1.82	0.78
65-69	m	TBI	Coma	cCMD	8	MCS-	33	39	13	-120	5	-0.67	-0.36
25-29	m	TBI	MCS+	cCMD	12	MCS-	35	43	8.5	-15	6	0.48	0.09
70-74	m	Stroke	UWS/VS	cCMD	15	MCS+	28	15	9.5	-45	6	0.25	0.29
30-34	f	Stroke	MCS-	cCMD	8	MCS+	43	43	18	-120	4	-1.32	0.02
65-69	m	Stroke	Coma	cCMD	17	MCS	24	21	10.5	-35	6	0.23	0.02
70-74	m	Stroke	UWS/VS	cCMD	15	MCS+	50	29	11	-100	7	0.12	0.14
20-24	m	TBI	Coma	cCMD	6	UWS/VS	13	34	7	40	7	1.15	-0.04
50-54	f	Stroke	UWS/VS	cCMD	4	UWS/VS	22	2	24	-275	2	-3.12	0.07
60-64	m	Stroke	UWS/VS	cCMD	6	UWS/VS	31	37	8	-15	6	0.52	0.1
15-19	m	TBI	MCS-	cCMD	9	MCS+	30	36	23	-220	3	-2.49	-0.13
50-54	f	Stroke	MCS+	cCMD	10	MCS+	32	29	7	20	7	1.05	-0.01
55-59	m	Stroke	MCS-	cCMD	12	MCS+	23	8	16	-105	5	-0.82	0.07
70-74	m	Stroke	MCS+	cCMD	23	EMCS	40	30	7	45	7	1.18	0.05
50-54	f	Hydroce-	MCS-	cCMD	23	EMCS	83	16	5	50	8	1.62	-0.01

		phalus with GRMS											
55-59	f	TBI	UWS/VS	cCMD	17	MCS+	21	16	4	65	8	1.78	0.03
40-44	m	TBI	Coma	cCMD	23	EMCS	24	18	8	80	8	1.54	0.01
50-54	m	TBI	Coma	cCMD	6	UWS/VS	33	34	13	-120	5	-0.67	0.12
40-44	m	TBI	Coma	cCMD	13	MCS+	24	26	8.5	-35	6	0.38	0.27
40-44	m	TBI	UWS/VS	cCMD	23	EMCS	50	18	7	-55	6	0.39	0.12
55-59	m	Anoxia	MCS-	cCMD	12	MCS-	21	38	18	-170	5	-1.31	-0.02
15-19	f	TBI	Coma	cCMD	7	MCS-	19	25	2.5	70	9	2.19	-0.04
40-44	m	TBI	Coma	DoC	0	Coma	6	17	29	-325	1	-4.03	0.06
30-34	m	Anoxia	Coma	cCMD	4	UWS/VS	23	43	9	20	6	0.63	-0.06
50-54	m	COVID-19 encepha-lopathy	UWS/VS	cCMD	23	EMCS	52	1	3	90	9	2.25	0.32
55-59	m	TBI	MCS-	cCMD	11	MCS	18	22	7	-55	6	0.39	0.03
60-64	f	Stroke	Coma	cCMD	10	MCS+	55	14	18.5	-20	4	-0.85	-0.43
70-74	m	Stroke	MCS+	cCMD	18	MCS+	24	11	7	15	6	0.75	0.22
65-69	m	TBI	MCS+	cCMD	13	MCS+	55	25	19	0	3	-1.05	0.3
45-49	f	TBI	MCS-	cCMD	19	EMCS	41	2	11	-110	5	-0.46	-0.17
25-29	f	TBI	UWS/VS	cCMD	9	MCS	20	21	11.5	-140	5	-0.66	0.12

Table S1. Demographic and clinical data, related to figure 4. TBI traumatic brain injury, GRMS Global Rostral Midbrain Syndrome, CRS-R Coma Recovery Scale Revised, UWS/VS unresponsive wakefulness syndrome/vegetative state, MCS-/+ minimally conscious state minus/plus, EMCS emergence of minimal conscious state, MBTr Motor Behaviour Tool revised, cCMD clinical cognitive motor dissociation, DRS Disability Rating Scale, ERBI Early Barthel Index, RLAS Rancho Los Amigos Level of Cognitive Functioning. Category on the Early Barthel Index range from -325 to 100, with 100 indicating complete functional independence; Category on the Disability Rating Scale range from 0 to 29, with 0 indicating absence of disability; Category on the Rancho Los Amigos Levels of Cognitive Functioning range from 1 to 10, with 10 indicating modified independent. Columns indicated as "at recording" indicate scores collected at the time of the EEG recording used to compute the PPS index.
